# ID1^high^/activin A^high^ glioblastoma cells contribute to resistance to anti-angiogenesis therapy through malformed vasculature

**DOI:** 10.1038/s41419-024-06678-7

**Published:** 2024-04-24

**Authors:** Sang-Hun Choi, Junseok Jang, Yoonji Kim, Cheol Gyu Park, Seon Yong Lee, Hyojin Kim, Hyunggee Kim

**Affiliations:** 1 Department of Biotechnology, Korea University, Seoul, 02841 Republic of Korea; 2 Institute of Animal Molecular Biotechnology, Korea University, Seoul, 02841 Republic of Korea; 3 MEDIFIC Inc, Hwaseong-si, Gyeonggi-do 18469 Republic of Korea

**Keywords:** CNS cancer, Cancer therapeutic resistance, Tumour angiogenesis

## Abstract

Although bevacizumab (BVZ), a representative drug for anti-angiogenesis therapy (AAT), is used as a first-line treatment for patients with glioblastoma (GBM), its efficacy is notably limited. Whereas several mechanisms have been proposed to explain the acquisition of AAT resistance, the specific underlying mechanisms have yet to be sufficiently ascertained. Here, we established that inhibitor of differentiation 1 (ID1)^high^/activin A^high^ glioblastoma cell confers resistance to BVZ. The bipotent effect of activin A during its active phase was demonstrated to reduce vasculature dependence in tumorigenesis. In response to a temporary exposure to activin A, this cytokine was found to induce endothelial-to-mesenchymal transition via the Smad3/Slug axis, whereas prolonged exposure led to endothelial apoptosis. ID1 tumors showing resistance to BVZ were established to be characterized by a hypovascular structure, hyperpermeability, and scattered hypoxic regions. Using a GBM mouse model, we demonstrated that AAT resistance can be overcome by administering therapy based on a combination of BVZ and SB431542, a Smad2/3 inhibitor, which contributed to enhancing survival. These findings offer valuable insights that could contribute to the development of new strategies for treating AAT-resistant GBM.

## Introduction

Glioblastoma (GBM) is a highly aggressive and heterogeneous form of primary brain cancer, with a median survival of 14 to 16 months [1–4]. At present, surgery, chemotherapy, and radiation therapy are the only available standard treatments for the care of patients with GBM [5, 6]. Furthermore, more than 90% of patients with GBM experience tumor relapse, with a median post-recurrence survival of less than 12 months [7, 8]. Consequently, there is a pressing need to develop new treatment approaches based on a comprehensive understanding of the pathogenesis of GBM.

The “angiogenic switch”, one of the hallmarks of cancer, induces the transition of quiescent blood vessels to an angiogenic state, thereby facilitating the continual proliferation of neoplastic cells [9]. Several studies have shown that angiogenesis is a prerequisite for the progression of cancer, consequently identifying anti-angiogenesis therapy (AAT) as a promising strategy for eliminating cancer and improving survival. In this regard, bevacizumab (BVZ), the first Food and Drug Administration-approved AAT drug that neutralizes vascular endothelial growth factor (VEGF), has been established to be highly effective in treating several types of solid tumors [10, 11]. However, despite the theoretical potential of AAT, it has yet to be demonstrated to clinically benefit patients with GBM, and although BVZ has been approved for use in patients with recurrent GBM, the prognosis of these patients has not been significantly improved [12]. Consequently, to enhance treatment outcomes, it is essential to gain more comprehensive understanding of the mechanisms underlying AAT resistance in GBM.

Activin A, which belongs to the transforming growth factor- β (TGF-β) superfamily, elicits cellular and biological changes reminiscent of TGF-β, and induces cell-type dependent phenotypic alterations [13]. Endothelial-to-mesenchymal transition (EndoMT) is a prominent phenomenon induced by TGF-β in which endothelial cells (ECs) acquire mesenchymal features, resulting in loss of cell-cell junctions, increase in cell motility, and elevated vascular permeability [14]. However, although such alterations in ECs have been demonstrated to be correlated with the progression of cancer and to confer resistance to diverse therapeutic modalities, details of the mechanisms underlying activin A-mediated changes in ECs and resistance to treatment have yet to be sufficiently determined.

Inhibitor of differentiation 1 (ID1), which has a helix-loop-helix structure that forms heterodimers with transcription factors, thereby inhibiting their function, has been shown to be highly expressed in GBM and identified as a key factor for maintaining features of glioma stem cells (GSCs) [15]. However, although ID1 is known to regulate intracellular signaling mechanisms in GSCs, its effect on the tumor microenvironment is unknown. Recent studies have suggested that the cells surrounding cancer cells promote malignancy and resistance to therapy. Therefore, it is necessary to investigate ID1-mediated changes in the tumor microenvironment. In this study, we elucidated the mechanism by which ID1 alters endothelial characteristics and confers resistance to AAT.

## Results

### ID1 is associated with vascular abnormality in GBM

**Fig. 1:**
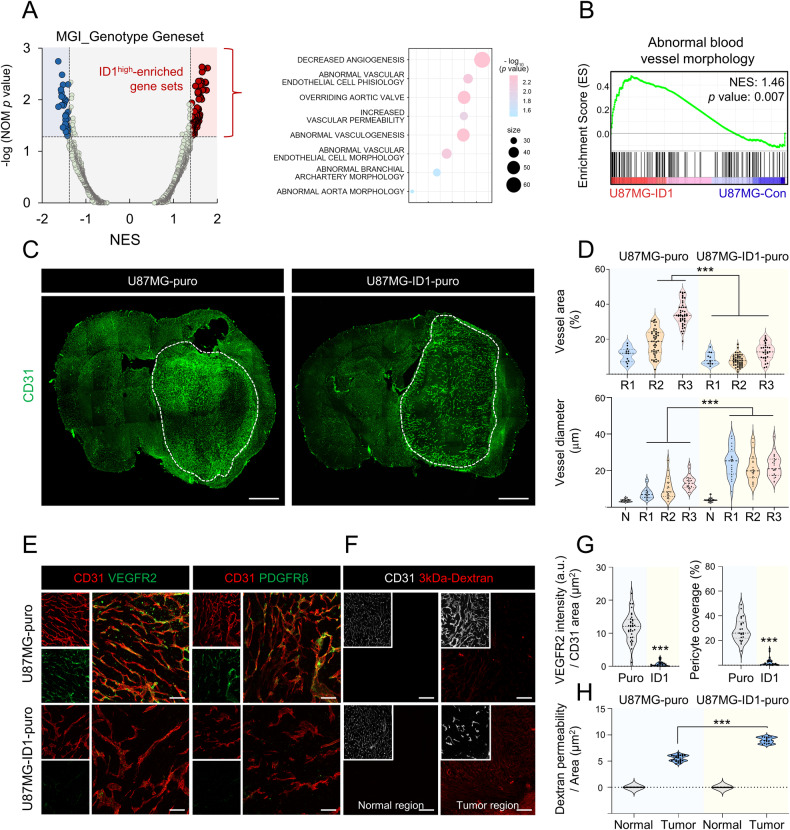
ID1 is related to abnormal vascularity in GBM. **A** Volcano plot visualizing the GSEA results of RNA-sequencing data from ID1-overexpressing U87MG cells. **B** GSEA revealed abnormal blood vessel morphology signature enrichment in ID1-overexpressing U87MG cells. **C** Whole brain images of CD31^+^ vessels in mice grafted with control and ID1-overexpressing tumors (Green; CD31). Scale bar, 1000 µm. **D** Quantification of the vessel area (%) and vessel diameter (µm) related to Fig. 1C (N, normal region; R1, tumor core region; R2, tumor core-marginal intermediate region; R3, tumor marginal region). *n* = 9–49 per group. ***P < 0.001 by unpaired *t* tests. **E** Immunofluorescence analysis of VEGFR2 intensity and pericyte coverage in mice grafted with control and ID1-overexpressing tumors (left panel: Green; VEGFR2, Red; CD31, right panel: Green; PDGFRβ, Red; CD31). Scale bar, 50 µm. **F** Immunofluorescence analysis of dextran permeability in mice grafted with control and ID1-overexpressing tumors (White; CD31, Red; 3 kDa-TRITC-dextran). Scale bar, 50 µm. **G** Quantification of the VEGFR2 intensity (a.u.) and pericyte coverage (%) related to Fig. 1E. *n* = 25–35 per group. ***P < 0.001 by unpaired *t* tests. **H** Quantification of the dextran permeability (a.u.) related to Fig. 1F. *n* = 16–25 per group. ***P < 0.001 by unpaired *t* tests.

In a previous study, we observed necrotic regions in ID1-overexpressing tumors, thereby indicating a potential correlation between ID1 and the vasculature [15]. To investigate the relationship between ID1 and the abnormal vasculature, we performed RNA-sequencing of ID1-overexpressing cells (Supplementary Fig. 1A). Subsequently, RNA-sequencing data was subjected to gene set enrichment analysis (GSEA), which revealed a pronounced enrichment of terms related to an aberrant vasculature (Fig. 1A, B). Moreover, a similar result was observed when the transcriptome acquired from a single-cell RNA-sequencing study with GBM was divided into ID1^high^ and ID1^low^ groups based on the expression of ID1 [16] (ID1^high^, average value > 1, false discovery rate (FDR) value < 0.05; ID1^low^, average value < −1, FDR value < 0.05) (Supplementary Fig. 1B). To validate the results obtained from our bioinformatics analysis, we performed mouse intracranial grafting of ID1-overexpressing cells and control cells and examined the effects on blood vessel structure. The analysis was based on the division of tissues into three regions according to tumor size (N, normal region; R1, tumor core region; R2, tumor core-marginal intermediate region; R3, tumor marginal region) (Supplementary Fig. 1C). Compared with the control tumors, the ID1-overexpressing tumors exhibited a notable decrease in the blood vessel area in the R2 and R3 regions, while no significant difference was observed in the R1 region. In contrast, we observed an increase in the thickness of blood vessels in the ID1-overexpressing tumors (Fig. 1C, D and Supplementary Fig. 1D, E). A variation in blood vessel thickness is associated with an aberrant vasculature, whereas decreased vessel areas may indicate the formation of necrotic regions. Interestingly, while hypoxic regions were observed only in the core of control tumors, ID1-overexpressing tumors exhibited hypoxic regions throughout the tumor (Supplementary Fig. 1F, G). Additionally, we validated the expression of VEGFR2, and assessed pericyte coverage and vascular permeability. Compared with the control tumors, the vasculature of ID1-overexpressing tumors exhibited low VEGFR2 expression, low pericyte coverage, and high dextran leakage (Fig. 1E–H). Collectively, the interior of the ID1-overexpressing tumors showed hypovasculature and aberrant vascular anatomy.

### ID1-upregulated activin A suppresses tube formation

**Fig. 2:**
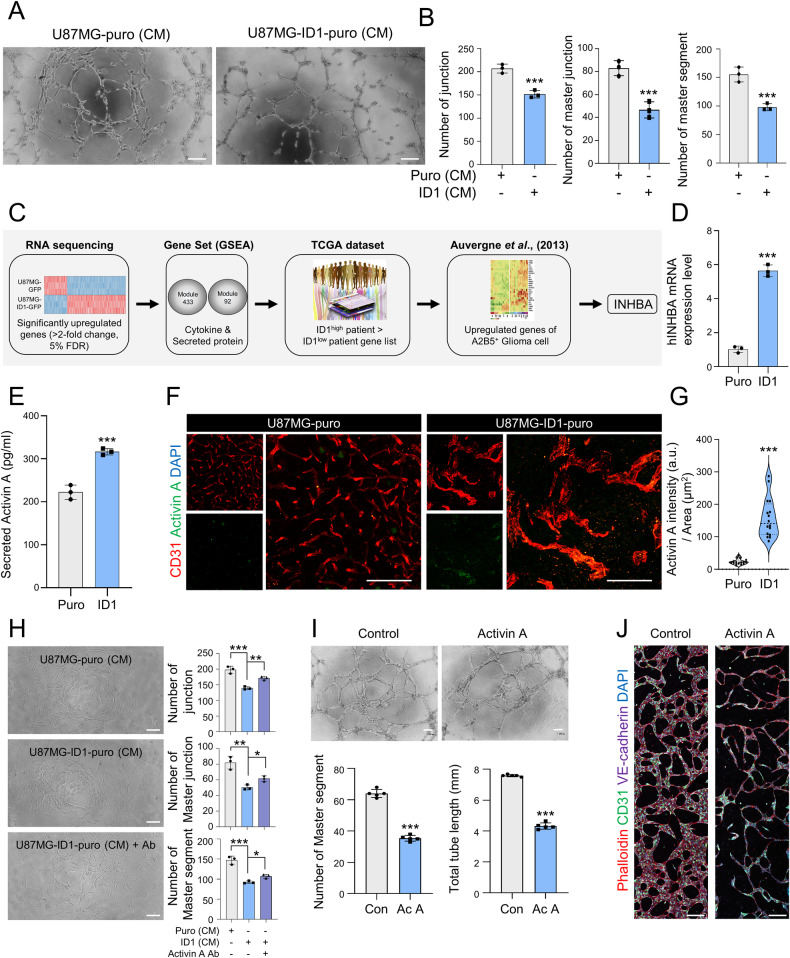
Activin A upregulated by ID1 suppresses tube formation ability. **A** Tube formation ability of HUVECs with conditioned medium obtained from control and ID1-overexpressing U87MG cells. Scale bar, 100 µm. **B** Quantification of the tube formation assay results shown in Fig. 2A. *n* = 3. ***P < 0.001 by unpaired *t* tests. **C** Flow chart showing four merged datasets to identify the factor induced by ID1. **D** The mRNA level of *INHBA* in control and ID1-overexpressing U87MG cells. *n* = 3. ***P < 0.001 by unpaired *t* tests. **E** Secretory levels of activin A in control and ID1-overexpressing U87MG cells measured by ELISA. *n* = 3. ***P < 0.001 by unpaired *t* tests. **F** Immunofluorescence analysis of activin A intensity in mice grafted with control and ID1-overexpressing tumors (Green; Activin A, Red; CD31, Blue; DAPI). Scale bar, 50 µm. **G** Quantification of the activin A intensity (a.u.) related to Fig. 2F. *n* = 16. ***P < 0.001 by unpaired *t* tests. **H** Tube formation ability of HUVECs in conditioned medium obtained from control and ID1-overexpressing U87MG cells and anti-activin A neutralizing antibodies. Quantification of the number of junctions, master junctions, and master segments. Scale bar, 100 µm. *n* = 3. ***P < 0.001, **P < 0.01, and *P < 0.05 by unpaired *t* tests. **I** Tube formation ability of HUVECs treated with activin A (10 ng/mL) at 5 h. Quantification of the number of master segments and total tube length. Scale bar, 100 µm. *n* = 3. ***P < 0.001 by unpaired *t* tests. **J** Immunofluorescence analysis of tube formation in a 3D-vasculogenesis microchip model (Red; Phalloidin, Green; CD31, Purple; VE-cadherin, Blue; DAPI). Scale bar, 200 µm.

To investigate the modulatory effects of ID1-regulated factors on ECs, we assessed the effects of conditioned medium (CM) on tube formation. Compared with the group treated with CM derived from control U87MG cells, we detected less-pronounced tube formation in the group treated with the CM derived from ID1-overexpressing cells (Fig. 2A, B). This result suggests that the paracrine effect of proteins secreted by ID1-overexpressing cells influences ECs. Next, we combined four types of publicly available data (i. upregulated genes of ID1-overexpressing U87MG cells, >2-fold, FDR < 0.05. ii. Module 92: secreted protein, *n* = 149 and Module 433: Cytokines and growth factors, *n* = 61. iii. data in which gene expression of patients with ID1^high^ was higher than that in patients with ID1^low^ with GBM was obtained from The Cancer Genome Atlas. iv. upregulated genes of A2B5^+^ glioma cells, ref. [17]). On the basis of this analysis, *INHBA* encoding activin A was identified as a factor that could potentially contribute to a reduction in tube formation (Fig. 2C). The activity of activin A can be modified by certain antagonists (*FST, INHA*, and *CFC1*) and negative regulator (*TGFBR3*). Hence, to exert its effect, *INHBA* must exhibit contrasting expression patterns. The expression patterns of *INHBA*, its antagonists, and negative regulator were compared based on reference to the Ivy GBM Atlas Project database (http://glioblastoma.alleninstitute.org/), which includes transcriptome data obtained for seven anatomical structures of patients with GBM. The levels of *ID1* and *INHBA* were higher in the hyperplastic blood vessel region, whereas compared with other regions, the levels of antagonists and negative regulator were lower compared to other regions (Supplementary Fig. 2A). Subsequently, we examined the level of expression of *INHBA* in ID1-overexpressing cells. Compared with the control cells, the mRNA level of *INHBA* was approximately 6-fold higher, and the secreted activin A level was 120 pg/mL higher in ID1-overexpressing cells (Fig. 2D, E). Furthermore, activin A expression was significantly higher in ID1-overexpressing tumors than in control tumors (Fig. 2F, G). The correlation between these *ID1* and *INHBA* was also confirmed in the results of transcriptomic analysis of various patients with GBM (Supplementary Fig. 2B). We subsequently used an anti-activin A neutralizing antibody to examine the effects of inhibiting ID1-upregulated activin A activity on tube formation upon exposure to CM. As a consequence of activin A neutralization, the activation of Smad3 was suppressed, thereby alleviating the inhibition of tube formation (Fig. 2H and Supplementary Fig. 2C). In addition, the tube formation ability was reduced in the presence of activin A alone (Fig. 2I). To further assess the effect of activin A on tube formation, we used a 3D microchip model designed to more closely mimic blood vessels in vivo, which accordingly revealed an absence of normal vessel development in response to treatment with activin A (Fig. 2J). Collectively, these findings provide evidence to indicate that the ID1-induced secretion of activin A inhibits tube formation via a paracrine effect.

### Transient exposure to activin A induces EndoMT in ECs via the activin A/Smad3/Slug axis

In terms of functionality, the function of activin A is similar to that of TGF-β, and, depending on the duration of exposure, can elicit contrasting effects, notably the induction of EndoMT or apoptosis [18]. Our examination of the transient effects of activin A on ECs revealed that short-term exposure to activin A results in the downregulation of EC-related genes, whereas we detected an upregulation of *SNAI2*, which encodes Slug, a transcription factor implicated in the induction of EndoMT (Supplementary Fig. 3A). Given that it has been established that TGF-β induces EndoMT via the Smad pathway, we investigated whether activin A similarly induces EndoMT via this pathway, and accordingly found that activin A promoted increases in the phosphorylation of Smad3 and the expression of mesenchymal markers, Slug, and nuclear Slug (Supplementary Fig. 3B, C).

Slug, a factor implicated in various diseases, is recognized for its capacity to induce EndoMT and promote the migration and invasion of ECs [19]. Activin A promoted the formation and rearrangement of stress fibers, which are representative features of migrating cells (Supplementary Fig. 3C, D). Subsequently, we directly evaluated EC migration and invasion in response to treatment with activin A, and observed increases not only in the invasive capacity but also in the migratory behavior of ECs (Supplementary Fig. 3E, F). Furthermore, we validated that the increased migration ability induced by activin A is dependent on Slug (Supplementary Fig. 3G, H). These findings indicated that the activin A/Smad3/Slug axis triggered EndoMT and subsequently enhanced the migratory capability of ECs.

### Slug-induced EndoMT contributes to an increase in vascular permeability

**Fig. 3:**
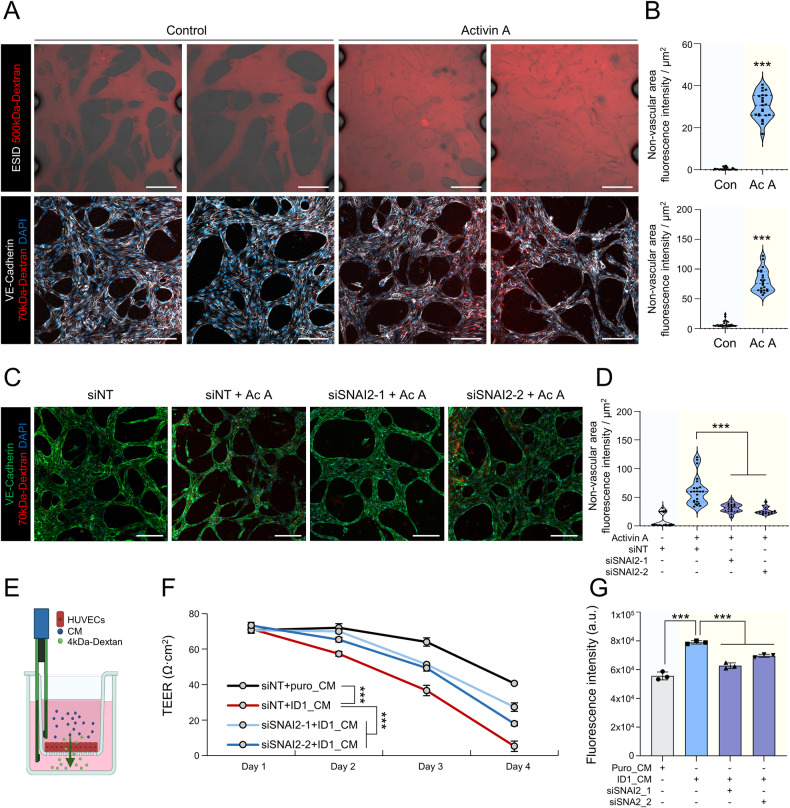
EndoMT induced by Smad3/Slug axis promotes vascular permeability. **A** Live imaging of vascular permeability in a 3D-vasculogenesis microchip model (ESID; HUVECs, Red; 500kDa-TRITC-dextran) with activin A (10 ng/mL), (upper images). Immunofluorescence analysis of vascular permeability in 3D-vasculogenesis microchip model (White; VE-cadherin, Red; 70kDa-TRITC-dextran, Blue; DAPI) with activin A (10 ng/mL). **B** Quantification of dextran permeability (a.u.). Scale bar, 100 µm. *n* = 23. ***P < 0.001 by unpaired *t* tests. **C** Immunofluorescence analysis of vascular permeability in a 3D-vasculogenesis microchip with activin A (10 ng/mL). (Green; VE-cadherin, Red; 70kDa-TRITC-dextran, Blue; DAPI). Scale bar, 100 µm. **D** Quantification of dextran permeability (a.u.). Scale bar, 100 µm. *n* = 19–23 per group. ***P < 0.001 by unpaired *t* tests. **E** Experimental scheme of the modified TEER assay. **F** TEER values of siNT- and siSNAI2-HUVECs treated with conditioned medium obtained from control and ID1-overexpressing U87MG cells. *n* = 3. ***P < 0.001 by unpaired *t* tests. **G** Quantification of the fluorescence intensity of 4kDa-FITC-dextran flowing in the bottom chamber. *n* = 3. ***P < 0.001 by unpaired *t* tests.

EndoMT is a crucial physiological process involved in vascular development. Nevertheless, it also plays a significant role in pathogenesis, serving as an exacerbating factor in cardiovascular diseases and several types of cancer [20, 21]. Notably, EndoMT is closely associated with hyperpermeability, a characteristic of the tumor vasculature [22]. To determine whether activin A plays a role in the induction of vascular permeability, we utilized a 3D microchip capable of forming a vascular network and perfused dextran-conjugated tetramethylrhodamine (TRITC). Dextran leakage from blood vessels increased upon activin A exposure, as observed in both live imaging and fixed imaging experiments (Fig. 3A, B and Supplementary Movies 1, 2). To verify whether this phenomenon was induced by the Smad3/Slug axis, we assessed vascular permeability following the knockdown of *SNAI2* (Supplementary Fig. 4). The knockdown of *SNAI2* alleviated the activin A-induced increase in vascular permeability (Fig. 3C, D). This result was confirmed by using a transepithelial electrical resistance assay (Fig. 3E–G). Consequently, activin A induced EndoMT via the Smad3/Slug axis, ultimately leading to increases in vascular permeability.

### ID1 contributes to a resistance to anti-VEGF therapy

**Fig. 4:**
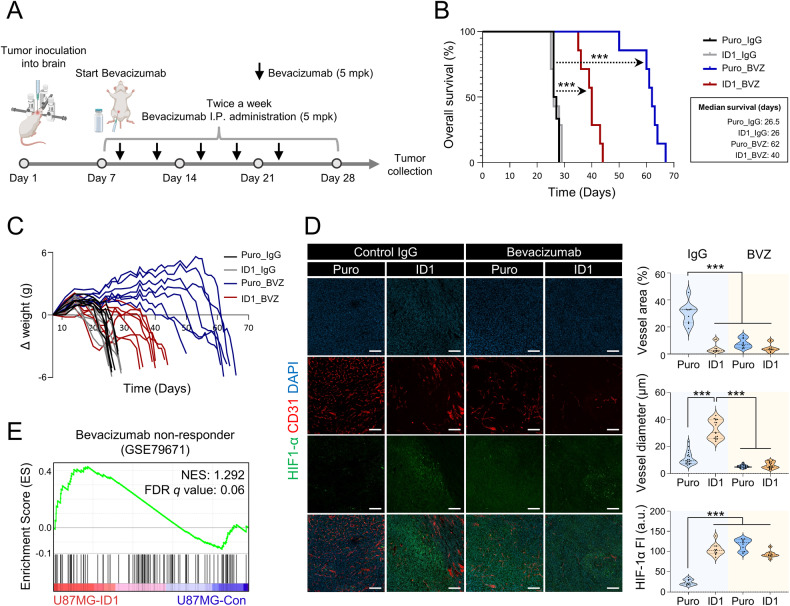
ID1-overexpressing tumors exhibit anti-VEGF therapy resistance. **A** Experimental scheme for the treatment of the GBM intracranial graft mouse model with bevacizumab (BVZ, 5 mg/kg, mpk). **B** Kaplan–Meier plots showing the survival rates of the GBM intracranial graft mouse model treated with BVZ. *n* = 6–7 per group. *P* values were obtained using Log-rank (Mantel-Cox) test. ***P < 0.001. **C** Bodyweight of the GBM intracranial graft mouse model. *n* = 6–7 per group. **D** Immunofluorescence analysis of the GBM intracranial graft mouse model treated with BVZ. Scale bar, 100 µm. *n* = 9–21 per group. ***P < 0.001 by unpaired *t* tests. **E** Single-sample GSEA was performed using RNA-sequencing data from ID1-overexpressing U87MG cells to identify BVZ non-responders among patients with GBM.

BVZ has been shown to be ineffective in improving the survival of patients with GBM [12]. Several mechanisms that contribute to BVZ resistance have been suggested, with the most prominent being a decreased reliance on VEGF for the development and maintenance of blood vessels, and the presence of cancer cells adapted to hypoxic environments. These findings suggest that in ID1-overexpressing tumors, ECs may exhibit decreased reliance on VEGF through EndoMT, while cancer cells are likely to adapt to hypoxia due to their hypovascular characteristics. Thus, we evaluated resistance to BVZ in a mouse model bearing intracranial graft GBM. 5 mpk (mg/kg) of BVZ were administered twice a week, starting on the 10^th^ day after cancer cells inoculation (Fig. 4A). Both normal IgG-treated groups (control and ID1-overexpressing mice) died within 25–29 days post cancer cells inoculation. BVZ-treated mice with ID1-overexpressing tumors died within 35–44 days. Notably, BVZ treatment was more effective in prolonging the survival of control mice compared with ID1-overexpressing mice (Fig. 4B, C). Furthermore, histological analysis revealed that compared with mice in the normal IgG-treated group, there were reductions in the area and diameter of vessels and an increase in cancer cell migration ability in the BVZ-treated mice (Fig. 4D and Supplementary Fig. 5A). These findings are consistent with observation in clinical patients. Next, to validate its clinical relevance, we classified BVZ-treated patients with GBM as either responders or non-responders, and on the basis of single-sample GSEA, we found that the non-responders were characterized by an enrichment of the ID1-overexpressing transcriptome (Fig. 4E).

Interestingly, multiple apoptotic vessels were observed in the ID1-overexpressing tumors (Supplementary Fig. 5B). To determine whether this phenomenon was associated with activin A, a vasculogenesis and angiogenesis model were formed in a 3D microchip and ECs were treated with activin A for 10 days. The group treated with activin A showed increased expression of cleaved caspase 3 compared with the untreated group (Supplementary Fig. 5C, D). These findings explain the hyperpermeability and hypovascular anatomical features of the vessel within the ID1-overexpressing tumors. Hence, ID1 contributes to anti-VEGF therapy resistance.

### SB431542 abolishes ID1-induced BVZ resistance in an intracranially grafted-GBM mouse model

**Fig. 5:**
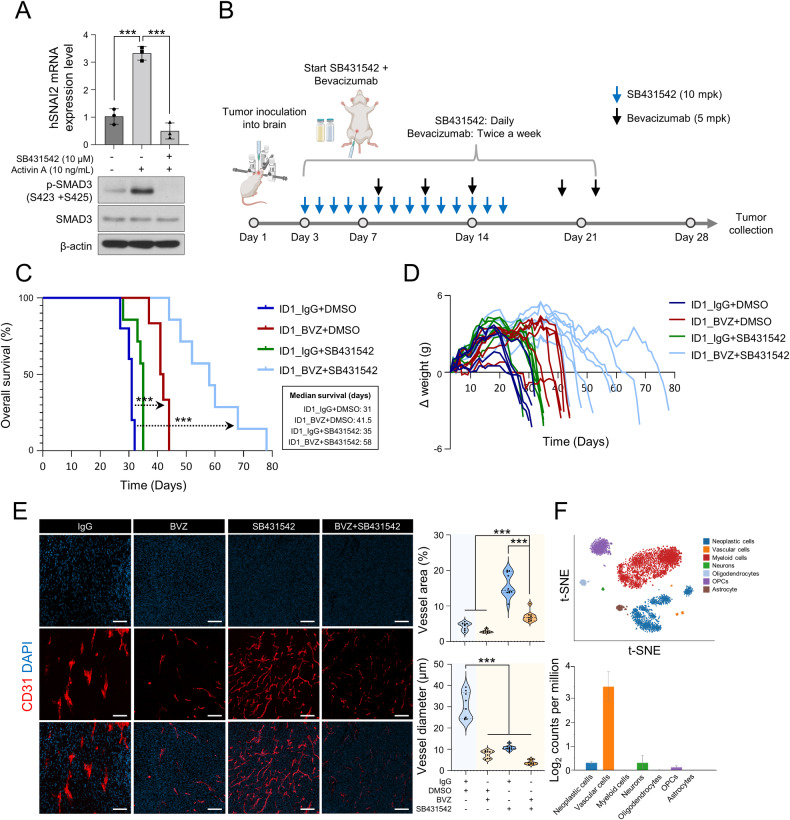
Combination therapy with SB431542 and BVZ overcomes BVZ-resistance in ID1-overexpressing tumors. **A** Western blot analysis of p-Smad3, Smad3, and β-actin in HUVECs treated with activin A and SB431542 (10 µM). mRNA levels of *SNAI2* in HUVECs treated with activin A (10 ng/mL) alone or in combination with SB431542. *n* = 3. ***P < 0.001 by unpaired *t* tests. **B** Experimental scheme for the treatment of the GBM intracranial graft mouse model with BVZ (5 mpk) and SB431542 (10 mpk). **C** Kaplan–Meier plots showing the survival rates of the GBM intracranial graft mouse models treated with BVZ and SB431542. *n* = 5–7 per group. *P* values were obtained using Log-rank (Mantel-Cox) test. ***P < 0.001. **D** Bodyweight of the GBM intracranial graft mouse model. *n* = 5–7 per group. **E** Immunofluorescence analysis of the GBM intracranial graft mouse model treated with BVZ and SB431542 (Red; CD31, Blue; DAPI). Quantification of the vessel area (%) and vessel diameter (µm). Scale bar, 100 µm. *n* = 8–10 per group. ***P < 0.001 by unpaired *t* tests. **F** t-SNE plots of cells from patients with GBM. The mRNA levels of *SNAI2* in various cell types. Data were obtained from www.gbmseq.org.

**Fig. 6:**
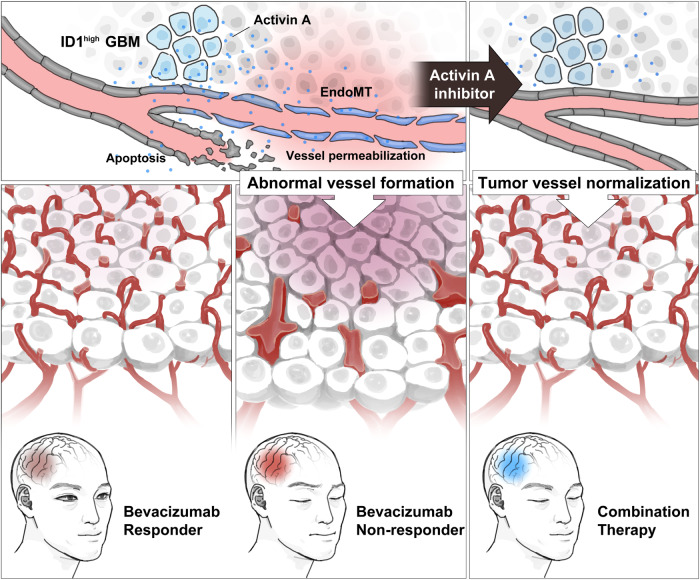
Inhibition of activin A/Slug axis overcomes BVZ resistance. The schematic diagram showing the BVZ resistance caused by ID1/activin A/Slug axis and therapeutic strategy in GBM.

SB431542 inhibits TGF-β/Smad3 signaling by targeting ALK4, 5, and 7 (Activin receptor-like kinase receptors). Thus, we investigated whether SB431542, a drug commonly used to inhibit epithelial-to-mesenchymal transition, could suppress activin A-induced EndoMT. The results showed that SB431542 treatment led to decreased expression of *SNAI2*, which is upregulated by activin A in ECs (Fig. 5A). However, SB431542 did not affect the proliferation of ID1-overexpressing cells (Supplementary Fig. 6). Next, we assessed the therapeutic efficacy of the simultaneous administration of SB431542 and BVZ in ID1-overexpressing tumors. SB431542 was administered daily for two weeks, whereas BVZ was administered using the previous regimen (Fig. 5B). The SB431542-treated group did not exhibit a significant difference in survival compared with the control group. However, the SB431542/BVZ combination-treated group showed a median survival extension of more than two weeks compared with the group treated with BVZ monotherapy (Fig. 5C, D). Interestingly, hypovascularity improved with SB431542 treatment, as evidenced by an increase in the vascular area and a decrease in vessel diameter. Furthermore, in the combination-treated group, hypovascularity was alleviated compared to that in the control group and the group treated with BVZ monotherapy (Fig. 5E). Subsequently, the selective upregulation of *SNAI2* in ECs present in patients with GBM was validated using single-cell RNA-sequencing data obtained by ref. [23] (Fig. 5F). In conclusion, the administration of SB431542 could represent an effective strategy for overcoming the resistance to BVZ induced by ID1 (Fig. 6).

## Discussion

This study presents the mechanism underlying the resistance to AAT caused by the dual functions of activin A on ECs, which are upregulated in GBM. Specifically, we demonstrated that temporal exposure to activin A induced EndoMT in ECs via the Smad/Slug axis. The ECs that undergo EndoMT were found to be characterized by enhanced migratory behavior and increased vascular permeability, thereby providing evidence to indicate that blood vessels become dysfunctional and less reliant on VEGF, which ultimately contributes to AAT resistance. Moreover, numerous studies have shown that EndoMT can impact cancer malignancy by modulating the tumor microenvironment, including the secretion of cytokines and direct interactions with cancer cells [24, 25]. Therefore, characterization and unbiased transcriptomic analysis of ECs within tumors should be performed in the future.

Previous studies have reported that prolonged exposure to TGF-β, which shares functional similarity to activin A, can induce apoptosis in ECs. Moreover, it has been established that whether TGF-β induces either pro-survival or pro-apoptotic signaling in ECs is dependent on the presence of VEGF. When exposed to TGF-β alone, the p38MAPK isoform p38b promotes EC survivability, whereas in contrast, p38a induces apoptosis when ECs are exposed to both VEGF and TGF-β [26]. Similar shifts in signaling have been observed in the case of activin A. EndoMT and apoptosis in ECs are induced depending on the tumor region. In this regard, it can be speculated that apoptotic regions are characterized by specific concentrations of VEGF that are associated with active angiogenesis, whereas conversely, the ECs that undergo EndoMT are primarily observed in the core-marginal region. Thus, both phenomena may occur based on the spatial distribution of VEGF in ID1-overexpressing tumors.

The mechanisms underlying the resistance to AAT remain unclear. Although this study suggests that the scattered hypoxic regions in ID1-overexpressing tumors could potentially contribute to AAT resistance, the characteristics of these regions that may confer resistance to cancer cells are not yet comprehensively understood. Interestingly, when only activin A was overexpressed, it showed no resistance to BVZ (Supplementary Fig. 7A–C). These findings suggest that not only activin A but also ID1 are crucial in conferring resistance to BVZ, indicating that BVZ could have an impact on cancer cells. According to several studies, inhibiting the signaling of VEGF/VEGF receptor 2 (VEGFR2) in U87MG cells leads to a decrease in cell growth and clonal proliferation [27, 28]. In fact, we found that compared with control cells, ID1-overexpressing cells are characterized by reductions in the expression of VEGFA and VEGFR2. In addition, exposure to a hypoxic environment was found to result in a gradual reduction in the expression of VEGFR2 over time, whereas we detected an increase in the expression of *INHBA* (Supplementary Fig. 7D–F). On the basis of these observations, we thus speculate that BVZ could have an effect on cancer cells, and that ID1-overexpressing cells may have reduced dependence on VEGF/VEGFR2 signaling, potentially contributing to an attenuation of the efficacy of BVZ. Furthermore, this process could be amplified by the hypoxic environment within ID1-overexpressing tumors.

Hypervascularization is a prominent feature of GBM, and although the blood vessels commonly observed in patients with GBM were found to be similar in appearance to those in control tumors, the vasculature of ID1-overexpressing tumors was established to have distinct characteristics. This was more like the vasculature observed in recurrent GBM compared with primary GBM. Indeed, our RNA-sequencing analysis of ID1-overexpressing cells revealed transcriptome similarity to recurrent GBM rather than primary GBM (Supplementary Fig. 7G). Given that BVZ is currently administered as first-line therapy for patients with recurrent GBM and that most patients eventually develop resistance to this treatment, our study holds particular significance for patients with recurrent GBM. In addition, the ID1-overexpressing tumor exhibiting increased vessel diameter, hyperpermeability, and abundance of hypoxic regions, may provide significant clinical insight into the observed resistance to BVZ in a cohort of 69 patients with recurrent GBM. This is of relevance given that tumors with similar vessel characteristics show limited response to BVZ [29].

Although we demonstrated the mechanisms underlying AAT resistance using in vitro and in vivo models, the clinical relevance of these findings remains limited. The transcriptomes of publicly available datasets of patients with GBM were analyzed; however, it is necessary to construct a cohort of patients with GBM resistant to AAT for further analysis.

Moreover, this study could not elucidate the mechanism of *INHBA* regulation by ID1. Elucidating the mechanism of ID1 in the regulation of *INHBA* is challenging owing to its structural features. Since ID1 lacks a DNA-binding domain, it cannot directly regulate transcription by binding to DNA. However, ID1 interacts with other proteins to inhibit their function. Therefore, identifying the binding partner of ID1 is critical for understanding signal transduction pathways. In this regard, 560 candidate proteins that could potentially interact with ID1 have been identified based on in silico analysis, and a pooled CRISPR/Cas9 system that can be used to specifically inhibit candidate proteins has been developed. It is accordingly anticipated that these approaches will make a valuable contribution to enhancing our current understanding of the mechanisms underlying the signaling between ID1 and *INHBA*.

Our study has two significant technical limitations. The first limitation is that the hypothesis can only be demonstrated using U87MG cells. Because GBM is extremely heterogeneous, it is crucial to validate the hypothesis across multiple cell lines, and the overexpression and knockdown experiments must be performed concurrently. However, the availability of cell lines suitable for studying the relationship between ID1 and BVZ resistance is limited. To investigate resistance to BVZ, cell lines must meet three criteria. (i) the existing blood vessels within the tumor should exhibit a high dependence on VEGF, (ii) the tumor’s growth should be reliant on vasculature, and (iii) the efficacy of the BVZ must be present. Furthermore, to confirm the association with ID1, cells with both low and high endogenous expression of ID1 were required. Unfortunately, we found U87MG cells that met three criteria and had low expression of ID1, but no cell lines with high expression of ID1. Therefore, if additional cell lines with high expression of ID1 are established in the future, further validation will be necessary. The second limitation is that SB431542, which is used to treat BVZ resistance, is not a selective activin A inhibitor. SB431542 inhibits the TGF-β type 1 receptor, which suppresses signals from other TGF-β superfamily members, including TGF-β1 and Nodal. Therefore, the SB431542 used in this study may not only inhibit the function of activin A. Nevertheless, based on our findings, it seems that the efficacy of SB431542 appears to be independent of the inhibition of signals from TGF-β1 and Nodal. TGF-β1 is known as a pro-angiogenic factor [30–33]. If the signals were inhibited by SB431542, angiogenesis should have been suppressed. However, Fig. 5E shows that the number of blood vessels increased when SB431542 was administered alone. Nodal is known to promote the growth of glioblastoma cells and inhibiting it has been shown to suppress angiogenesis [34, 35]. However, according to Fig. 5E and Supplementary Fig. 6A, SB431542 had no effect on glioblastoma cell growth, and the pattern of angiogenesis is consistent with that of TGF-β1. Furthermore, RNA sequencing data revealed no difference in TGF-β1 and Nodal expression levels between ID1-overexpressing and non-overexpressing cells. Although we conducted an experiment to suppress the signal of activin A using SB431542, if the selective inhibitor of activin A is developed later, it must be used to investigate the relationship between activin A and BVZ resistance.

The ID family is composed of ID1, ID2, ID3, and ID4, with TGF-β known to regulate their expression. Interestingly the expression of ID genes is reduced by TGF-β in non-tumoral cells [36]; in contrast, TGF-β elicits an upregulation of ID gene expression in specific cancer cells. In breast cancer, the TGF-β induced upregulation of ID1 promotes mesenchymal-epithelial transition, playing a crucial role in the colonization stage of metastasis [37]. In glioma-initiating cell, pivotal for cancer initiation and recurrence, the treatment of a TGF-β type 1 receptor inhibitor led to a reduction in the expression levels of ID1 and ID3 [38]. These findings indicate the involvement of various ID genes associated with TGF-β in the malignancy progression of cancer. Therefore, it is important to elucidate the association between TGF-β and ID genes, and furthermore, each factor could potentially serve as a novel therapeutic target in developing new treatment strategies. Currently, various TGF-β inhibitors are undergoing clinical trials in diverse cancer types. Additionally, in the case of ID inhibitors, AGX51 inhibitor capable of simultaneously inhibiting the entire ID family are demonstrating promising efficacy [39]. Therefore, we anticipate providing specific evidence of the signaling pathway of TGF-β and ID genes in further study, aiming to achieve a more comprehensive understanding of the intricate signaling pathway.

To summarize, this study revealed the mechanism underlying resistance to AAT in the treatment of GBM and suggested that inhibition of activin A could be an efficient therapeutic strategy to overcome BVZ resistance in GBM.

### Supplementary information

Supplementary Information

Supplementary Movie 1

Supplementary Movie 2

Supplementary Table

Uncropped WB images

## Data Availability

The datasets used and/or analyzed during the current study are available from the corresponding author on reasonable request.
