# Supplementary material for: ID1high/activin Ahigh glioblastoma cells contribute to resistance to anti-angiogenesis therapy through malformed vasculature
Source: Cell Death Dis. 2024 Apr 24;15(4):292. doi: 10.1038/s41419-024-06678-7 (PMC11043395; doi:10.1038/s41419-024-06678-7)
Supplement: Supplementary file 1 — Supplementary Information [file 41419_2024_6678_MOESM1_ESM.pdf]

Supplemental information

ID1^{high}/activin A^{high} glioblastoma cell contributes to resistance of anti-angiogenesis therapy through malformed vasculature

Sang-Hun Choi, Junseok Jang, Yoonji Kim, Cheol Gyu Park, Seon Yong Lee, Hyojin Kim, and Hyunggee Kim

Supplemental Material and methods

Supplemental Figures 1-7 and legends

Supplemental Movie 1-2 legends

Material and methods

Cell culture and culture conditions

The human glioma cell line U87MG (*TP53* wild-type, mutant *PTEN*, *p14ARF/p16*del) was purchased from the American Type Culture Collection (ATCC, Virginia, USA, Cat. HTB-14) and authenticated using short tandem repeat profiling. The U87MG cells were cultured in high-glucose Dulbecco's modified Eagle's medium (DMEM; Hyclone, Logan, UK, Cat. SH30243.01) supplemented with 10% fetal bovine serum (Hyclone, Cat. SH30919.03), 1% penicillin/streptomycin (Hyclone, Cat. SV30010), 2 mM L-glutamine (Hyclone, Cat. SH30034.01), and 50 μg/mL gentamicin (Cellgro, NY, USA, Cat. MT-61-098-RF) at 37°C, 5% CO_2, and 95% humidity. In experiments with $CoCl_2$ (300 μM; Sigma-Aldrich, Cat. 232696), the cells were treated with $CoCl_2$ for 24 h and 48 h.

Human umbilical vein endothelial cells (HUVECs) were purchased from Lonza (Basel, Switzerland, Cat. CC-2517). The HUVECs were cultured on 0.2% gelatin-coated plates (Sigma-Aldrich, Cat. G1890) in endothelial cell growth medium-2 (EGM-2; Lonza, MO, USA, Cat. CC-3162). In experiments with SB431542 (10 μM; Sigma-Aldrich, Cat. S4317), the cells were pretreated with the inhibitor for 30 min before activin A treatment. All experiments were performed using cells passaged for a maximum of five times.

Plasmid construction and virus infection

U87MG cells were infected with lentivirus obtained from HEK293FT cells (Thermo Fisher Scientific, MA, USA, Cat. R70007) transfected with lentiviral vectors (pLL-CMV-GFP, pLL-CMV-ID1-GFP, pLL-CMV-Puro, pLL-CMV-ID1-Puro, and pLL-CMV-INHBA-puro) and packaging vectors (third generation: pMDLg/pRRE, pRSV-Rev, and pMD2.G). The lentivirus

was incubated with cells in the presence of polybrene (6 μg/mL, Sigma-Aldrich, Cat. H9268) for 24 h, followed by a media change. When generating the U87MG-Puro, U87MG-ID1-Puro, and U87MG-INHBA-puro cell lines, a pLL-CMV-GFP plasmid was utilized as an infection positive control. Puromycin (Clontech, Cat. 631306) treatment commenced from the point of GFP expression following infection. Puromycin selection was carried out with a concentration of 2 μg/mL on the first day, 3 μg/mL on the third day, and 5 μg/mL on the fifth day. The selection process was terminated when all the cells infected with the GFP plasmid were eliminated in puromycin. When generating the U87MG-GFP and U87MG-ID1-GFP cell lines, cells were cultured for one week post lentivirus infection, followed by sorting only GFP^+ cells using FACS (Fluorescence activated cell sorting, BD Biosciences, BD FACSAria II). After the first sorting, cells were cultured for an additional two weeks, followed by a second round of sorting. All viruses were tittered (Takara, Cat. 631235) immediately after production, and U87MG cells were infected at a multiplicity of infection of 1.

Short interfering RNA (siRNA) transfection

For knockdown of *SNAI2*, HUVECs (1.25×10^5 cells) were seeded onto a 60-mm cell culture plate. After 48 h, siRNA transfection was performed using ScreenFect A siRNA transfection reagent with a concentration of 10 nM (Wako Pure Chemical Industries, Osaka, Japan, Cat. 293-73201). A media change was performed 6 h post-transfection. The siRNA sequences for human *SNAI2* were as follows: 5'-CCAUUCUGAUGUAAAGAAAUA-3'and 5'-GAAUGUCUCUCCUGCACAA-3'.

Western blot analysis

Protein expression was analyzed by western blotting. Briefly, cell extracts were prepared using radioimmunoprecipitation assay lysis buffer (150 mM sodium chloride, 1% NP-40, 0.1% sodium dodecyl sulfate (SDS), and 50 mM Tris (pH 7.4)) containing 1 mM β-glycerophosphate, 2.5 mM sodium pyrophosphate, 1 mM sodium fluoride, 1 mM sodium orthovanadate, and a protease inhibitor (Roche, Basel, Switzerland, Cat. 11836170001). The protein concentration in the lysate was quantified using Bradford assay reagent (Bio-Rad), following the manufacturer's instructions. Proteins were resolved using SDS-polyacrylamide gel electrophoresis. The resolved proteins were then transferred onto a polyvinylidene fluoride membrane (Millipore, MA, USA, Cat. IPVH00010). The membrane was blocked with 5% non-fat milk and incubated with the following antibodies at the indicated dilutions: anti-ID1 (1:1000; Biocheck, Cat. BCH-1/195-14-50), anti-Smad3 (1:1000; Cell Signaling, MA, USA, Cat. 9523), anti-p-Smad3 (1:1000; Abcam, Cambridge, UK, Cat. ab52903), anti-FN1 (1:1000; Abcam, Cat. Ab6328), anti-α-SMA (1:1000; Sigma-Aldrich, Cat. A2547), anti-Slug (1:1000; Cell Signaling, Cat. 9585), anti-HIF-1α (1:500; Abcam, Cat. ab51608), anti-VEGFR2 (1:1000; Cell Signaling, Cat. 2479), and anti-β-actin antibodies (1:10000; Santa Cruz Biotechnology, TX, USA, Cat. sc-47778). Next, the membrane was incubated with horseradish peroxidase-conjugated anti-IgG secondary antibodies (Pierce Biotechnology). Immunoreactive signals were visualized using the SuperSignal West Pico chemiluminescent substrate (Pierce Biotechnology, MA, USA, Cat. 34580).

Quantitative real-time polymerase chain reaction (qRT-PCR)

The mRNA levels of the target genes were determined using qRT-PCR. Briefly, total RNA was isolated from cells using the QIAzol lysis reagent (QIAGEN, Hilden, Germany, Cat. 79306), following the manufacturer's instructions. Next, the isolated RNA (1 μg) was incubated

with 1 U of DNase I (RNase-free; Thermo Fisher Scientific, Cat. EN0525) at 37°C for 30 min. To inactivate DNase I, the samples were incubated with 50 mM ethylenediaminetetraacetic acid at 65°C for 10 min. DNase I-treated RNA was used as a template to synthesize complementary DNA (cDNA) using the RevertAid First Strand cDNA synthesis kit (Thermo Fisher Scientific, Cat. K1622), following the manufacturer's instructions. The qRT-PCR analysis was performed using Takara Bio TB Green Premix Ex Taq (Takara, Kusatsu, Japan, Cat. RR420A) and CFX096 (Bio-Rad, Hercules, CA, USA) under the following thermocycling conditions: 95°C for 30 s (initial denaturation), followed by 45 cycles of 95°C for 5 s (annealing) and 60°C for 30 s (elongation). The expression of each target gene was normalized to that of GAPDH. The relative expression levels of the target gene were quantified using the 2−ΔΔCq method. The sequence regarding the primers used in this article can be found in Supplemental Table 1.

Transepithelial electrical resistance (TEER) value

To measure the TEER values of the monolayers, HUVECs (5×10^4 cells) were seeded into transwell inserts (diameter, 6.5 mm; pore size, 5.0 μm; Corning, Cat. 3421). The insert was pre-coated with human plasma fibronectin (Sigma-Aldrich, Cat. F0895) in phosphate-buffered saline (PBS) for 2 h at 37°C in an incubator and air-dried for 45 min. At 1 h post-seeding, the insert was transferred to an empty well containing 850 μL EGM-2. The TEER value was measured one day after seeding using an ERS-2 epithelial volt-ohm meter (Millipore, Cat. MERS00002). STX3 electrodes were introduced into the apical and basolateral compartments of the inserts. For each experiment, the TEER value was measured using a cell-free transwell insert (blank). The final TEER values were multiplied by the surface area of the inserts and expressed as Ωcm^2. At the end of the experiment, FITC-dextran (500 ng/mL in EGM-2; Sigma-

Aldrich, Cat. 46944) was transferred to the upper chamber, and the samples were incubated for 10 min. The fluorescence intensity of the flowing FITC-dextran was measured using a multidetection microplate reader (Hidex Sense; HIDEX) [40].

Preparation of CM

For CM collection, U87MG-Puro and U87MG-ID1-Puro cells (5×10^5 cells) were seeded in a 100-mm culture plate with DMEM/F12 (Hyclone, Cat. SH30023.01) containing 0.2% B27 (Gibco, NY, USA, Cat. 17504044), epidermal growth factor (20 ng/mL; R&D Systems, MN, USA, Cat. 236-EG-01M), basic fibroblast growth factor (20 ng/mL; R&D systems, Cat. 4114-TC), 1% penicillin/streptomycin, 2 mM L-glutamine, and 50 μg gentamicin (Cellgro, Cat. MT-61-098-RF). After 48 h, the CM was harvested and filtered using a 0.22-μm filter (Sartorius, Gottingen, Germany, Cat. 16534). To neutralize activin A, CM and neutralizing antibodies (3 μg/mL; R&D Systems, Cat. AF338) were incubated for 1 h before CM was treated on cells.

Tube formation assay

A 96-well flat bottom plate was pre-cooled on an ice rack and coated with 55 μL of ECMatrix (Millipore, Cat. ECM625) at 37°C for 1 h. HUVECs (1.5×10^4 cells) were seeded into the pre-coated 96-well plates in EGM-2 or CM obtained from U87MG-Puro or U87MG-ID1-Puro cell cultures. Representative images of tube formation were captured after 3 h using a microscope (Olympus, Cat. CKK53, magnification ×10). Image analysis was performed using the Image J software (v.1.52a, plugin; Angiogenesis Analyzer, http://imagej.nih.gov/ij/).

Enzyme-linked immunosorbent assay (ELISA)

To analyze the secreted levels of Activin A, the U87MG-Puro and U87MG-ID1-Puro cultures were centrifuged at 3500 rpm for 5 min to obtain CM. The CM was filtered through a 0.22-μm syringe filter (Sartorius, Cat. 16534) to remove the cells. The concentration of Activin A was quantified using a Quantikine ELISA kit (R&D Systems, Cat. DAC00B). The final activin A concentration was calculated by dividing the quantified activin A concentration by the total number of cells.

Invasion assay

An invasion assay was performed using transwell (diameter, 6.5 mm; pore size, 5.0 μm; Corning, Cat. 3421) coated with 1 mg/mL Cultrex (R&D Systems, Cat. 3432-005-01P). The Cultrex was allowed to solidify at 37°C for 4 h. Next, HUVECs (1.5×10^5 cells) suspended in 100 μl serum-free EGM-2 were seeded into the upper chamber, and 500 μl complete EGM-2 was added to the lower chamber. After 48 h, cells that did not migrate were removed, and the migrated cells were fixed and stained with crystal violet. The image was analyzed by Image J software (v.1.52a, http://imagej.nih.gov/ij/).

Wound healing assay

HUVECs (1.5×10^5 cells) were seeded in a 6-well plate pre-coated with 0.2% gelatin. The medium was replaced daily until a confluent monolayer was obtained. The cells were treated with mitomycin C (50 μg/mL; Sigma-Aldrich, Cat. M4287) for 2 h to inhibit cell proliferation. The cell monolayer was manually scratched using a 1000-μL plastic pipette tip. Next, the cells were washed twice with PBS and incubated with EGM-2. Images were captured every 24 h

using inverted microscope and the distance of wound was quantified using ImageJ software (v.1.52a, http://imagej.nih.gov/ij/).

Three-dimensional (3D) angiogenesis assay on a microchip

The microchip devices were purchased from AIM Biotech (MA, USA, Cat. DAX-1). Fibrinogen (11.11 mg/mL; Sigma-Aldrich, Cat. F8630) was incubated overnight in a 37°C water bath to achieve complete dissolution. Next, fibrinogen was filtered through a 0.22-µm filter and incubated with thrombin (100 U/mL; Sigma-Aldrich, Cat. T7201), aprotinin (3 U/mL; Sigma-Aldrich, Cat. A3428), and EGM-2 on ice racks. Vial 1 (fibrinogen 90 µL + aprotinin 10 µL) and Vial 2 (EGM-2 100 µL + thrombin 1 µL) solutions were prepared to fill the gel channel. A mixture of Vial 1 and Vial 2 solutions (1:1 ratio) was transferred into the gel channel (10 µL), and the samples were incubated in a 37°C incubator for 15 min. A cutting-yellow tip was inserted into the inlet of the media channel. Next, the samples were incubated with 15 µL of the coating solution (50 µg/mL fibronectin diluted in EGM-2, Sigma-Aldrich, Cat. F0895) at 37°C for 1 h. To flush out the coating solution, 70 µL of EGM-2 was added into one port and 50 µL of EGM-2 was added into the opposite port of the same media channel. Next, 65 µL of EGM-2 was added to both ports of the same media channel. The holder on which the chip was mounted tilted to 90°. An additional 20 µL of EGM-2 was added to one of the media channels to be seeded with the cells. A 10-µL pipette was used to transfer 10 µL of the HUVEC suspension (8×10^4 cells/10 µL). The samples were left undisturbed for 2 min. The same procedure was repeated for the other inlet. The holder was tilted to 90° in a 37°C incubator for 1 h. After moving the holder horizontally, the media (EGM-2 with VEGFA; 40 ng/mL, R&D Systems, Cat. 293-VE, sphingosine-1-phosphate 125 nM, Millipore, Cat. 860492P) was replaced once every 12 h.

Three-dimensional (3D) vasculogenesis assay on a microchip

Microchip devices were purchased from AIM Biotech (Cat. DAX-1). Fibrinogen (11.11 mg/mL; Sigma-Aldrich, Cat. F8630) was placed overnight in a 37°C water bath for complete dissolution. Next, fibrinogen was filtered through a 0.22-μm filter and incubated with thrombin (100 U/mL; Sigma-Aldrich, Cat. T7201), aprotinin (3 U/mL; Sigma-Aldrich, Cat. A3428), and EGM-2 on ice racks. Vial 1 (fibrinogen 90 μL + aprotinin 10 μL) and Vial 2 (EGM-2 with HUVECs 100 μL (4×10^4 cells/100 μL) + thrombin 1 μL) solutions were prepared to fill the gel channel. A mixture of Vial 1 and Vial 2 solutions (1:1 ratio) was transferred into the gel channel (10 μL), and the samples were incubated in a 37°C incubator for 15 min. A cutting-yellow tip was inserted into the inlet of the media channel. Next, 15 μL of EGM-2 was injected, and the samples were incubated at 37°C for 1 h. To flush out the hydration solution, 70 μL of EGM-2 was added into one port and 50 μL of EGM-2 was added into the opposite port of the same media channel. The media (VEGFA; 40 ng/mL, EGF; 20 ng/mL, bFGF 20ng/mL) was replaced once every 12 h.

Glioma intracranial graft mouse model

Female BALB/c Nu/Nu mice (Orient Bio, South Korea, 5 weeks of age) were anesthetized with Zoletil (19 mg/kg bodyweight) and Rompun (4.8 mg/kg bodyweight). U87MG-Puro (1×10^5), U87MG-ID1-Puro (1×10^5), and U87MG-INHBA-Puro (1×10^5) cells were transplanted into the caudate putamen of the brain (coordinates relative to the bregma; medial-lateral, +2 mm, anterior-posterior 0.5 mm, dorsal-ventral, -3 mm) using a stereotaxic injection device. i) Bevacizumab (5 mg/kg bodyweight; Roche) or bevacizumab vehicle control (5 mg/kg bodyweight; BioCell, Cat. BP0297) was intraperitoneally administrated 10 days after brain injection, twice a week, five times. The number of mice used in the experiment is as

follows (U87-Puro with IgG, n=6; U87-Puro with Bevacizumab, n=7; U87-ID1-Puro with IgG, n=7; U87-ID1-Puro with Bevacizumab, n=7). The number of mice used in the experiment is as follows (U87MG-Puro with IgG, n=5; U87MG-Puro with Bevacizumab, n=6; U87MG-INHBA-Puro with IgG, n=6; U87MG-INHBA-Puro with Bevacizumab, n=6). ii) Bevacizumab (5 mg/kg bodyweight; Roche), bevacizumab vehicle control (5 mg/kg bodyweight; BioCell, Cat. BP0297), SB431542 (10 mg/kg bodyweight; Selleckchem, TX, USA, Cat. S1067) diluted with PEG300 (Selleckchem, Cat. S6704), or DMSO (Sigma-Aldrich, Cat. D2650) as the SB431542 vehicle control (10 mg/kg bodyweight; Sigma-Aldrich, Cat. D2650). SB431542 was administered intraperitoneally daily for 3 days after brain injection for 2 weeks. Bevacizumab was intraperitoneally administrated after 8 days after brain injection twice a week, five times. The number of mice used in the experiment is as follows (U87MG-ID1-Puro with IgG, n=5; U87MG-ID1-Puro with Bevacizumab, n=6; U87MG-ID1-Puro with SB431542, n=7; U87MG-ID1-Puro with Bevacizumab and SB431542, n=7). Pimonidazole (60 mg/kg bodyweight; HPI, MA, USA, Cat. HP2-100) was injected intravenously to observe the hypoxic region immediately before the mice were euthanized. In this experiment, three mice were used for each group. To analyze vascular permeability, 3 kDa TRITC-conjugated dextran (2 mg/mL; Invitrogen, Cat. D3308) was injected intravenously 7 min before euthanasia. In this experiment, five mice were used for each group. The mice were anesthetized by intraperitoneal administration of avertin (250 mg/kg bodyweight; Sigma-Aldrich, Cat. T48402). The Korea University Institutional Animal Care & Use Committee approved the animal experiments, which were carried out in accordance with governmental and institutional guidelines as well as Korean regulations (approval no. KUIACUC-2022-0033). Mice were bred with under the following conditions: average temperature of 20°C-24°C; humidity of 45%-65%; circadian cycle, 12-h light/dark cycle. Mice received a continuous supply of food and water.

Fluorescence imaging

HUVECs were cultured on coverslips in 24-well plates for 1 d. Next, the cells were fixed with 4% PFA for 10 min, followed by washing four times with PBS, permeabilizing with 0.3% Triton X-100 (Sigma-Aldrich, Cat. T8787) in PBS for 10 min at room temperature and blocking with 3% bovine serum albumin (BSA; Millipore, Cat. 821006) in PBS for 1 h at room temperature. The cells were then incubated with the anti-CD31 antibodies (1:200; Thermo Fisher Scientific, Cat. IHC-00055) overnight at 4°C, followed by incubation with the Alexa Fluor 488-conjugated or Alexa Fluor 568-conjugated secondary antibodies (1:400; Invitrogen, Cat. A10042, A21202) and phalloidin-conjugated Alexa Fluor 568 antibodies (1:400; Invitrogen, Cat. A12379) for 2 h at room temperature. The nuclei were stained with DAPI (1 μg/mL; Sigma-Aldrich, Cat. D9542) for 5 min at room temperature. The image was analyzed by Image J software (v.1.52a, http://imagej.nih.gov/ij/).

For histological analysis, tumor-bearing brain tissues were fixed overnight in 4% PFA and dehydrated in 30% sucrose (Sigma-Aldrich, Cat. S0389) in PBS for 48 h. Next, the tissues were then embedded in the tissue freezing medium (Leica, Wetzlar, Germany, Cat. 3801480) for 3 h at −25°C. The frozen tissue blocks were cut into 40-μm thick sections. These sections were blocked with 3% BSA containing 0.3% Triton X-100 in PBS overnight at 4°C and incubated with the primary anti-CD31 (1:200; Merck, NJ, USA, Cat. MAB1398Z), anti-Activin A (1:50; R&D Systems, Cat. AF338), anti-Slug (1:100; Cell Signaling, Cat. 9585), anti-PDGFRβ (1:50; eBioscience, CA, USA, Cat. 14-1402-82), anti-cleaved CASP3 (1:200; Cell Signaling Cat. 9661), and anti-VEGFR2 (1:200; Cell Signaling, Cat. 2479) antibodies for 12 h at 4°C. After washing four times with PBS containing 0.1% Tween-20 (PBS-T), the sections were incubated with the Alexa Fluor 488-conjugated, Alexa Fluor 568-conjugated, or Alexa Fluor 647-conjugated secondary antibodies (1:400; Jackson Lab, Cat. 127-605-160) for 12 h at 4°C.

Nuclei were stained with DAPI (1 μg/mL) for 10 min at room temperature. The samples were then mounted with ProLongTM Gold Antifade Mountant (Invitrogen, MA, USA, Cat. p36930). To detect hypoxic areas, tissues were blocked with 3% BSA containing 0.3% Triton X-100 in PBS overnight at 4°C and incubated with the FITC-conjugated anti-pimonidazole antibodies (1:50) for 12 h at 4°C. The area of blood vessels and the diameter of blood vessels were determined by measuring the $CD31^+$ area within randomly selected regions of 0.4 mm^2. Pericyte coverage was quantified by assessing the degree of colocalization between $CD31^+$ area and $PDGFR\beta^+$ area within randomly selected tumor regions of 0.4 mm^2. The fluorescence intensity of VEGFR2 was measured on $CD31^+$ area within randomly selected tumor regions of 0.4 mm^2. The fluorescence intensity of 3 kDa-Dextran, HIF1-α, hydroxyprobe, and cleaved caspase 3 were measured within randomly selected regions of 0.4 mm^2. The image was analyzed by Image J software (v.1.52a, http://imagej.nih.gov/ij/).

For immunofluorescence analysis of microfluidic devices, 3D angiogenesis and 3D vasculogenesis models were established using HUVECs. The samples were fixed with 4% PFA for 20 min. The microfluidic device was washed four times with PBS, permeabilized with 0.3% Triton X-100 in PBS for 20 min at room temperature and blocked with 3% BSA in PBS for 2 h at room temperature. Next, the microfluidic device was incubated with the primary anti-VE-cadherin (1:200; Santa Cruz Biotechnology, Cat. SC9989), anti-cleaved CASP3 (1:200; Cell Signaling, Cat. 9661), and anti-CD31 (1:200; Bethyl Laboratory, TX, USA, Cat. IHC-00055) antibodies overnight at 4°C, followed by incubation with Alexa Fluor 488-conjugated or Alexa Fluor 647-conjugated secondary antibodies (1:400; Invitrogen, Cat. A21202, A31573) for 2 h at room temperature. Nuclei were stained with DAPI (1 μg/mL; Sigma-Aldrich, Cat. D9542) for 10 min at room temperature. The fluorescence intensity of cleaved caspase 3 in 3D angiogenesis model was measured within randomly selected regions of 0.8 mm^2 (The triangular shape in the microchip was adjusted to be positioned at the top of the image). The fluorescence

intensity of cleaved caspase 3 in 3D vasculogenesis model was measured within randomly selected regions of 1.6 mm^2 (The triangular shape in the microchip was adjusted to be positioned on either side of image). The image was analyzed by Image J software (v.1.52a, http://imagej.nih.gov/ij/).

For live cell imaging of the microfluidic device, a 3D vasculogenesis model was established using HUVECs. Next, 500 kDa TRITC-dextran (500 ng/mL; Sigma-Aldrich, Cat. 52914) was allowed to flow into the media channel. Images were immediately captured using a confocal laser scanning microscope (CLSM; Carl Zeiss, Cat. LSM800) in a live cell incubator (Chamlide, Cat. TC-FC5N).

For analysis of 70 kDa TRITC-dextran (500 ng/mL; Invitrogen, Cat. D1818), the medium containing 70 kDa TRITC-dextran was allowed to flow into the media channel for 10 min. The samples were washed thrice with PBS and subsequently stained as described above.

All images were captured using CLSM and ZEN acquisition software version 2018 [blue edition] at room temperature.

In silico analysis

To analyze the region-specific transcriptome profiles of patients with GBM, the data of fragments per kilobase of transcripts per million mapped were obtained from the Ivy Glioblastoma Atlas Project (Ivy GAP) website (http://glioblastoma.alleninstitute.org/). Regional information was divided into the following seven types: hyperplastic blood vessels in the cellular tumor (n=22), microvascular proliferation (n=28), leading-edge (n=19), infiltrating tumor (n=24), cellular tumor (n=111), perinecrotic zone (n=26), and pseudopalisading cells around necrosis (n=40). Relative gene expression levels were converted

to z-scores, and the mean ± SD value of the z-score was calculated. The gene expression profiles of patients with GBM, compiled using TCGA, were downloaded from cBioPortal (cbioportal.org). The RNA-seq data obtained by eBiogen were grouped based on changes in the expression of ID1 (higher than two-fold relative to the baseline value) to establish ID1-based differentially expressed genes by comparing the mean expression values (Student's t-test; P < 0.05, FDR 5%) (GEO: GSE182670) [41]. The data was obtained from GSE122871 [16], and cells were divided into two groups ($ID1^{high}$ and $ID1^{low}$ groups) based on the two-fold difference in the mean expression values (Student's t-test; P < 0.05). The transcriptomes of patients with GBM treated with bevacizumab were obtained from GSE79671 [42]. GSEA (version 20.2.1) and Single-sample GSEA (version 10.1.0) were conducted using algorithms on the GenePatterns website (cloud.genepattern.org) and gene sets related to mammalian phenotype annotations from the Mouse Genome Informatics (MGI) website (www.informatcis.jax.org).

To analyze the correlation between ID1 and INHBA, the transcriptomes of patients with GBM were obtained from GlioVis portal (gliovis.bioinfo.cnio.es). The data was analyzed by GraphPad Prism (Version 9.3.1) [43,44,45].

Statistical analysis

All statistical analyses were performed using an unpaired Student's t-test and one-way ANOVA, followed by a Bonferroni post hoc test. When comparing the two groups, significance was determined using the Student's t-test. Comparisons between multiple groups were performed using one-way ANOVA, followed by Bonferroni post hoc test. All statistical analyses were performed using GraphPad Prism (Version 9.3.1). Values of P<0.05 or P<0.01 were considered statistically significant for different experiments, as indicated in the figure

legends. Data are presented as the mean ± SEM.

Supplemental Figures

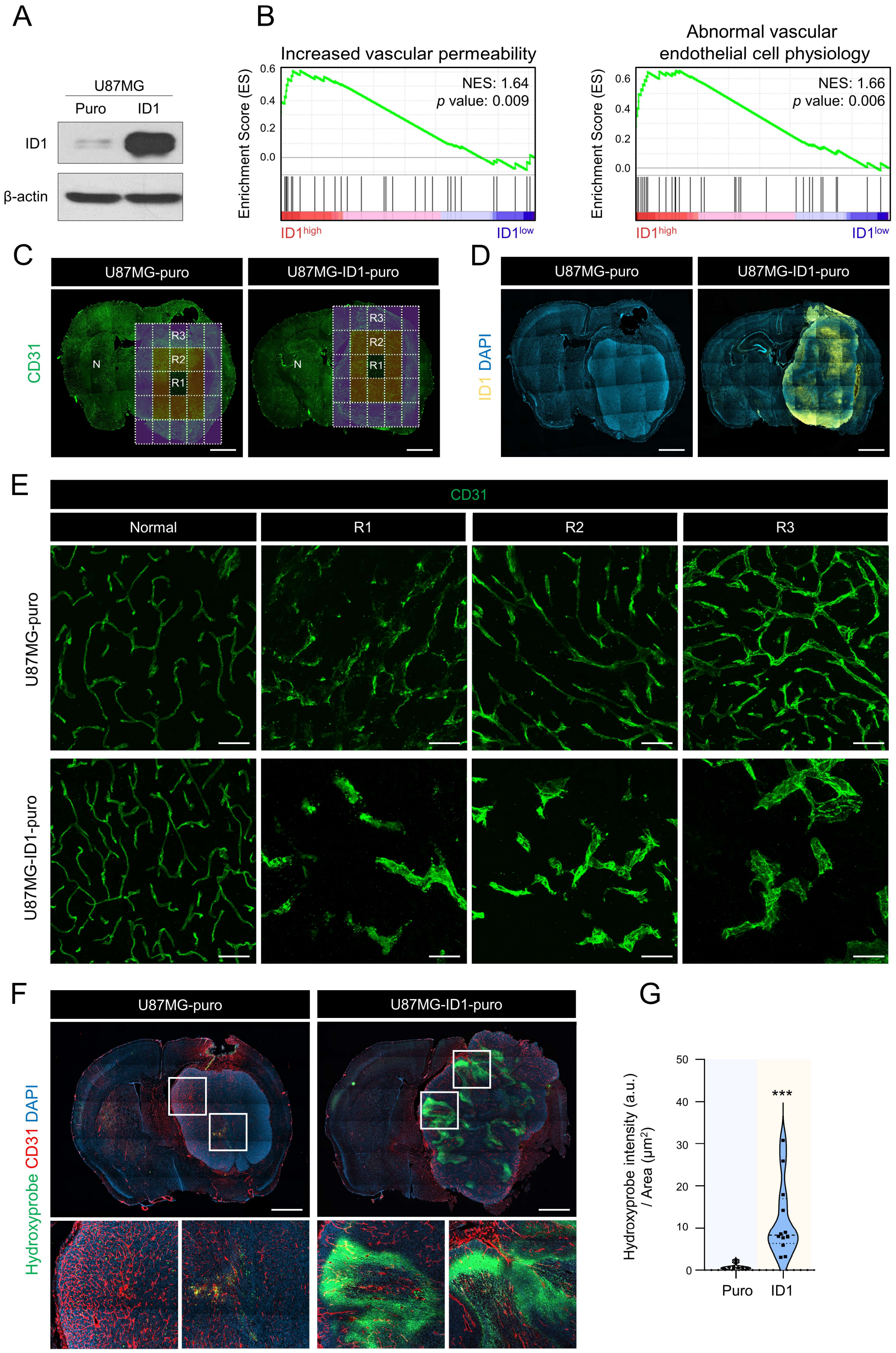

Supplemental Fig. 1 ID1 is associated with malformed vasculature in GBM.

(A) Western blot analysis of ID1 and β-actin in control and ID1-overexpressing U87MG cells.

(B) GSEA comparing the enrichment of $ID1^{high}$ and $ID1^{low}$ cells obtained from GSE122871 (based on the two-fold difference in the mean expression value, Student's t-test; $P < 0.05$)

(C) Compartmentalization method for quantification of vessel area (%) and diameter (μm). Scale bar, 1000 μm.

(D) Whole brain images of $ID1^{+}$ cancer cells in mice grafted with control and ID1-overexpressing tumors (Yellow; ID1, Blue; DAPI). Scale bar, 1000 μm.

(E) Immunofluorescence results showing $CD31^{+}$ vessels in different regions in mice grafted with control and ID1-overexpressing tumors (Green; CD31). Scale bar, 100 μm.

(F) Whole brain images of $hydroxyprobe^{+}$ regions in mice grafted with control and ID1-overexpressing tumors (Green; Hydroxyprobe, Red; CD31, Blue; DAPI). Scale bar, 1000 μm.

(G) Quantification of hydroxyprobe intensity in mice grafted with control and ID1-overexpressing tumor. n = 10, 12. $^{***}P < 0.001$. Data are presented as the mean ± SEM.

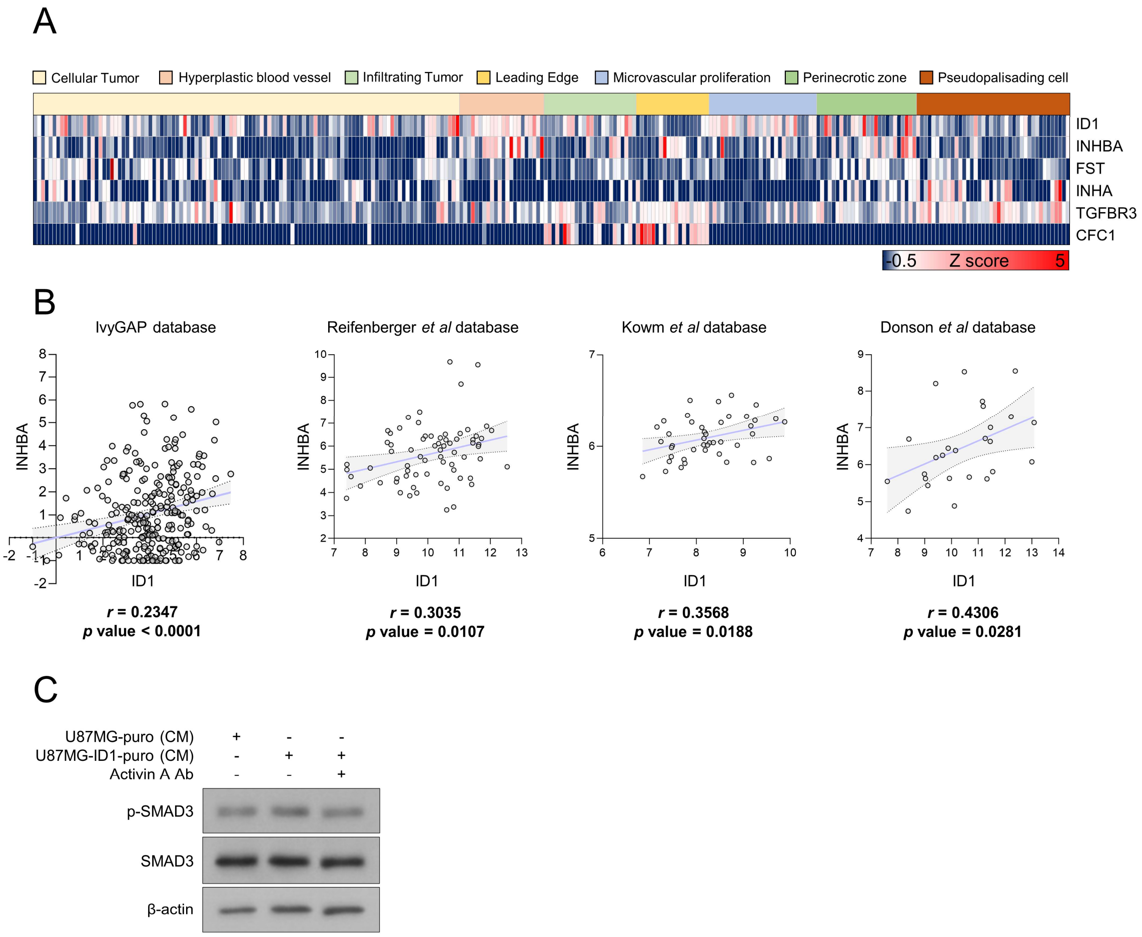

Supplemental Fig. 2 The expression of *INHBA* is upregulated in the hyperplastic blood vessel region in tissues obtained from patients with GBM.

(A) Analysis of the mRNA expression levels *ID1*, *INHBA*, its antagonists (*FST*, *INHA*, and *CFC1*), and negative regulator (*TGFBR3*) was performed using the Ivy Glioblastoma Atlas Project database (http://glioblastoma.alleninstitute.org/). Relative mRNA levels were converted to z-scores and depicted as a heatmap.

(B) Analysis of correlation between *ID1* and *INHBA* in publicly available transcriptome of patients with GBM. Transcriptome was obtained from GlioVis Portal (gliovis.bioinfo.cnio.es).

(C) Western blot analysis of p-Smad3, Smad3, and β-actin in HUVECs treated with conditioned medium obtained from control and ID1-overexpressing U87MG cells and anti-activin A neutralizing antibodies.

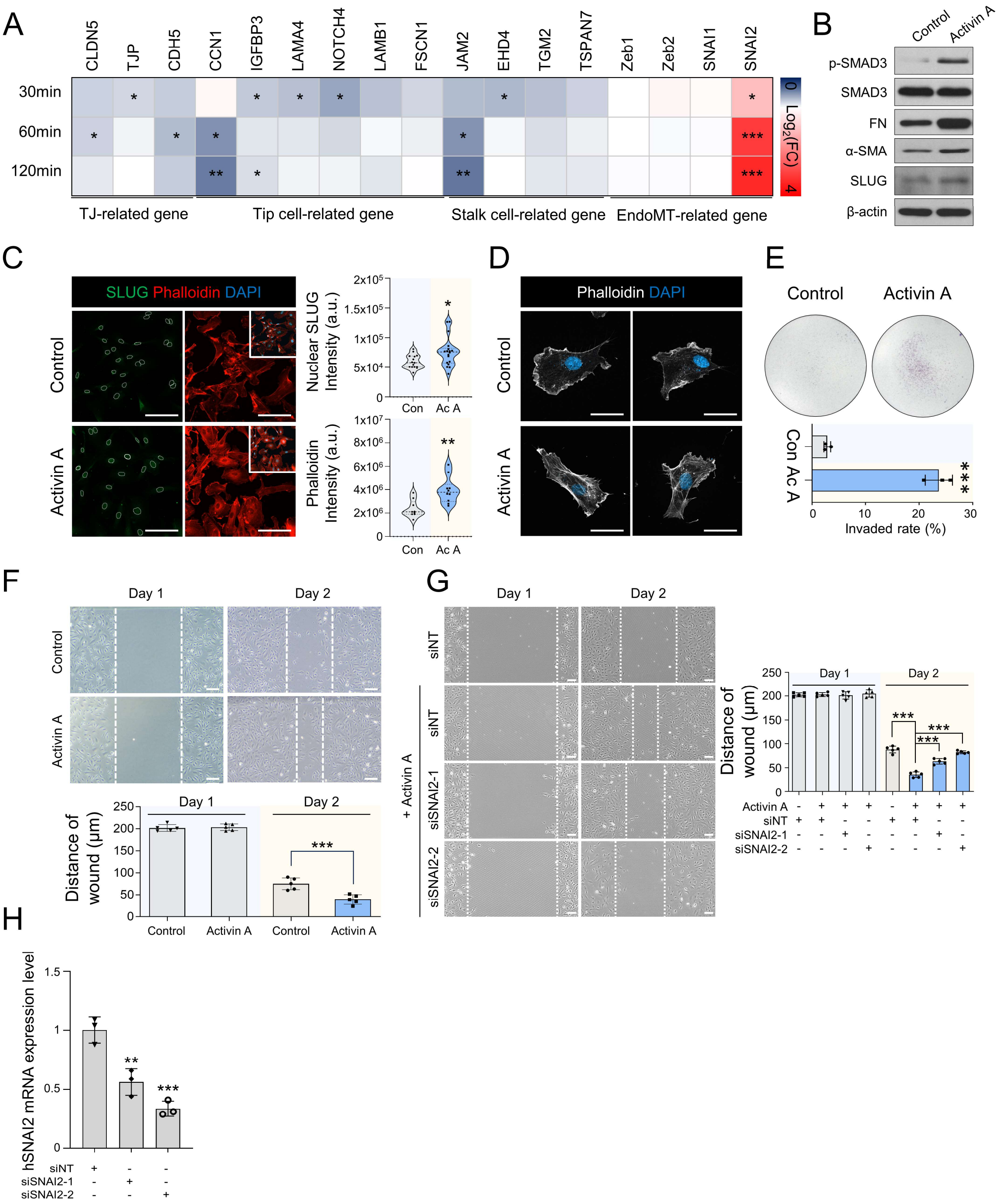
A
CLDN5
TJP
CDH5
CCN1
IGFBP3
LAMA4
NOTCH4
LAMB1
FSCN1
JAM2
EHD4
TGM2
TSPAN7
Zeb1
Zeb2
SNAI1
SNAI2
30min
60min
120min
0
Log2(FC)
4
TJ-related gene
Tip cell-related gene
Stalk cell-related gene
EndoMT-related gene
B
Control
Activin A
p-SMAD3
SMAD3
FN
α-SMA
SLUG
β-actin
C
SLUG Phalloidin DAPI
Control
Activin A
Nuclear SLUG Intensity (a.u.)
Phalloidin Intensity (a.u.)
Con
Ac A
D
Phalloidin DAPI
Control
Activin A
E
Control
Activin A
Con
Ac A
Invaded rate (%)
F
Day 1
Day 2
Control
Activin A
Distance of wound (μm)
Control
Activin A
Control
Activin A
G
Day 1
Day 2
siNT
+ Activin A
siNT
siSNAI2-1
siSNAI2-2
Distance of wound (μm)
Activin A
siNT
siSNAI2-1
siSNAI2-2
H
hSNAI2 mRNA expression level
siNT
siSNAI2-1
siSNAI2-2

Supplemental Fig. 3 EndoMT induced by activin A promotes endothelial invasion and migration ability.

(A) Heatmap showing the effect of activin A (10 ng/mL) on the mRNA expression of endothelial cell-related genes in HUVECs. n = 3. ***$P < 0.001$, **$P < 0.01$, and *$P < 0.05$ by unpaired t-tests.

(B) Western blot analysis of p-Smad3, Smad3, FN, α-SMA, Slug, and β-actin in HUVECs treated with activin A (10 ng/mL).

(C) Immunofluorescence analysis of Slug and phalloidin in HUVECs treated with activin A (10 ng/mL) (Green; Slug, Red; phalloidin, Blue; DAPI). Quantification of nuclear Slug and phalloidin intensity. Scale bar, 100 µm. n = 10-18 per group. **$P < 0.01$ and *$P < 0.05$ by unpaired t-tests.

(D) Immunofluorescence analysis of phalloidin in HUVECs treated with activin A (10 ng/mL). (White; phalloidin, Blue; DAPI). Scale bar, 20 µm.

(E) Invasion assay showing the effects of activin A (10 ng/mL) on HUVEC. n = 3. ***$P < 0.001$ by unpaired t-tests.

(F) Wound healing assay showing the effects of activin A (10 ng/mL) on HUVEC migration. Quantification of the wound distance (µm). Scale bar, 200 µm. n = 5. ***$P < 0.001$ by unpaired t-tests.

(G) Wound healing assay showing the effects of activin A (10 ng/mL) and *SNAI2* knockdown on HUVEC migration. Quantification of the wound distance (µm). Scale bar, 200 µm. n = 5. ***$P < 0.001$ by unpaired t-tests.

(H) The mRNA level of *SNAI2* in HUVECs with *SNAI2* knockdown. n = 3. *** $P < 0.001$ and **$P < 0.01$ by unpaired t-tests.

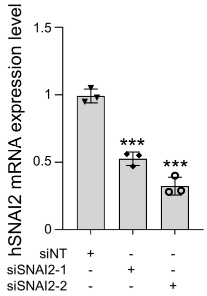

Supplemental Fig. 4 Knockdown of *SNAI2* in HUVECs related to Fig. 3.

The mRNA level of *SNAI2* in HUVECs with *SNAI2* knockdown. n = 3. *** $P < 0.001$ by unpaired t-tests.

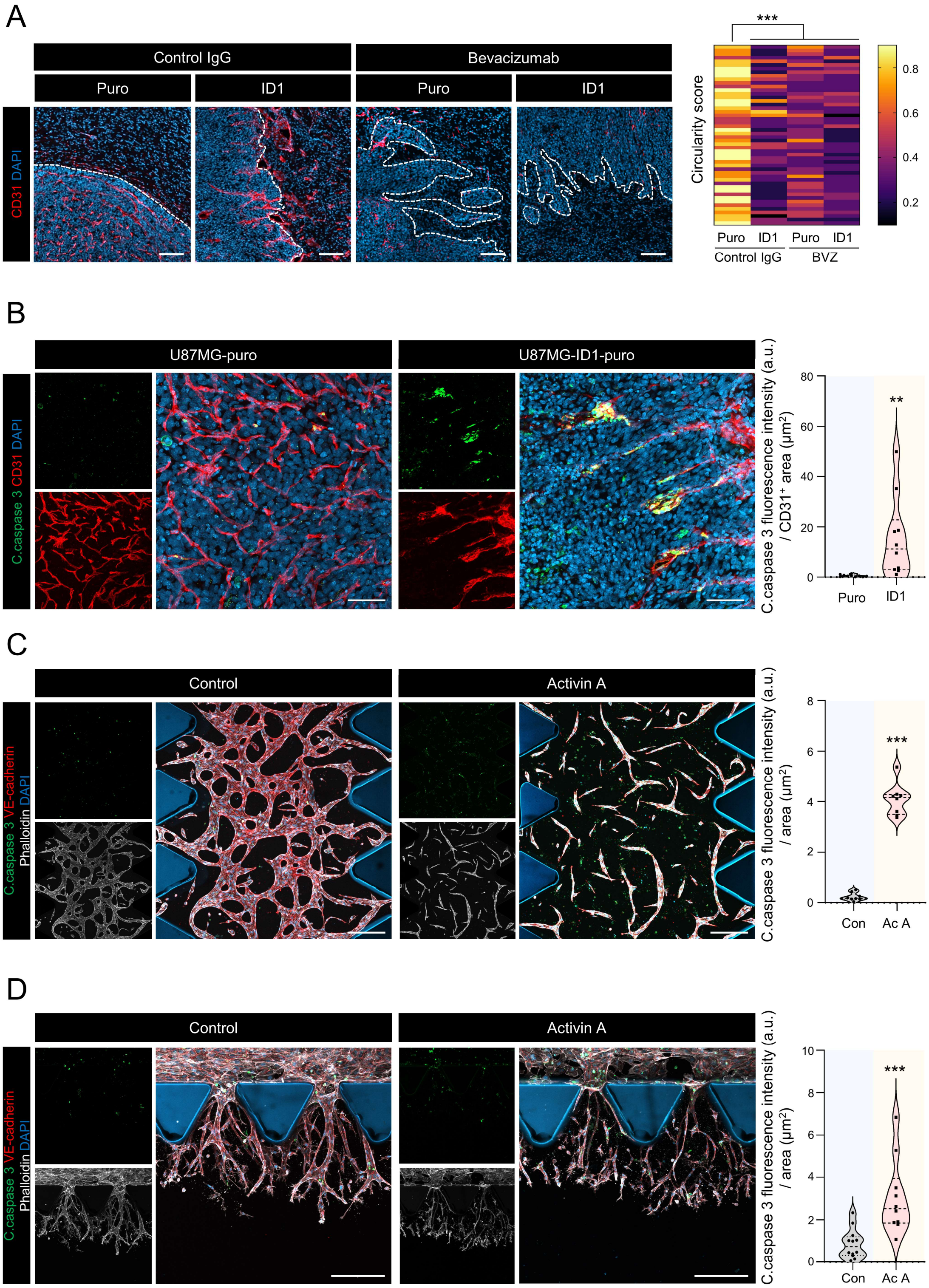
A
Control IgG
Bevacizumab
Puro
ID1
Puro
ID1
CD31 DAPI
***
Circularity score
0.8
0.6
0.4
0.2
Puro
ID1
Puro
ID1
Control IgG
BVZ
B
U87MG-puro
U87MG-ID1-puro
C.caspase 3 CD31 DAPI
C.caspase 3 fluorescence intensity (a.u.) / CD31⁺ area (µm²)
80
60
40
20
0
**
Puro
ID1
C
Control
Activin A
C.caspase 3 VE-cadherin Phalloidin DAPI
C.caspase 3 fluorescence intensity (a.u.) / area (µm²)
8
6
4
2
0
***
Con
Ac A
D
Control
Activin A
C.caspase 3 VE-cadherin Phalloidin DAPI
C.caspase 3 fluorescence intensity (a.u.) / area (µm²)
10
8
6
4
2
0
***
Con
Ac A

Supplemental Fig. 5 Histological changes after treatment with BVZ in GBM intracranial graft mouse model.

(A) Immunofluorescence analysis of CD31 and DAPI in mice grafted with control and ID1-overexpressing tumors treated with BVZ (Red; CD31, Blue; DAPI, White line; infiltration region). Quantification of circularity score. n = 50. Scale bar, 100 µm. ****P* < 0.001 by unpaired t-tests.

(B) Immunofluorescence analysis of endothelial apoptosis in mice grafted with control and ID1-overexpressing tumors (Green; Cleaved caspase3, Red; CD31, Blue; DAPI). Quantification of Cleaved caspase intensity (a.u.). n = 10. Scale bar, 100 µm. ***P* < 0.01 by unpaired t-tests.

(C) Immunofluorescence analysis of endothelial apoptosis in a 3D-vasculogenesis microchip model (Green; Cleaved caspase3, Red; VE-cadherin, White; phalloidin, Blue; DAPI). Quantification of Cleaved caspase 3 intensity (a.u.). n = 8. Scale bar, 200 µm. ****P* < 0.001 by unpaired t-tests.

(D) Immunofluorescence analysis of endothelial apoptosis in a 3D-angiogenesis microchip model (Green; Cleaved caspase3, Red; VE-cadherin, White; phalloidin, Blue; DAPI). Quantification of Cleaved caspase 3 intensity (a.u.). n = 12, 10. Scale bar, 200 µm. ****P* < 0.001 by unpaired t-tests.

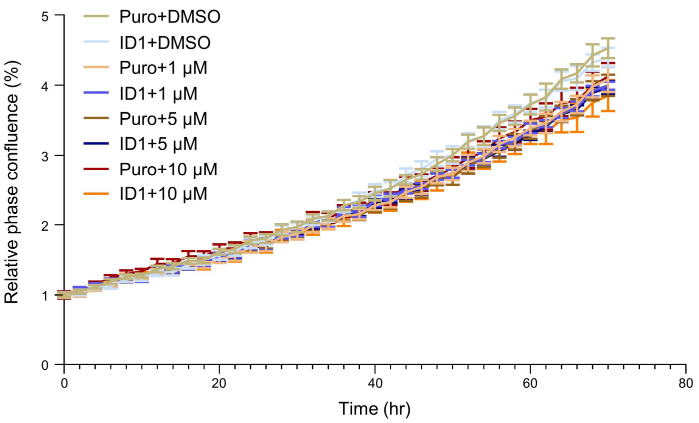

Supplemental Fig. 6 SB431542 does not affect ID1-overexpressing U87MG cells.

Cell proliferation assay of control and ID1-overexpressing U87MG cells treated with SB431542 using live cell imaging. n = 5.

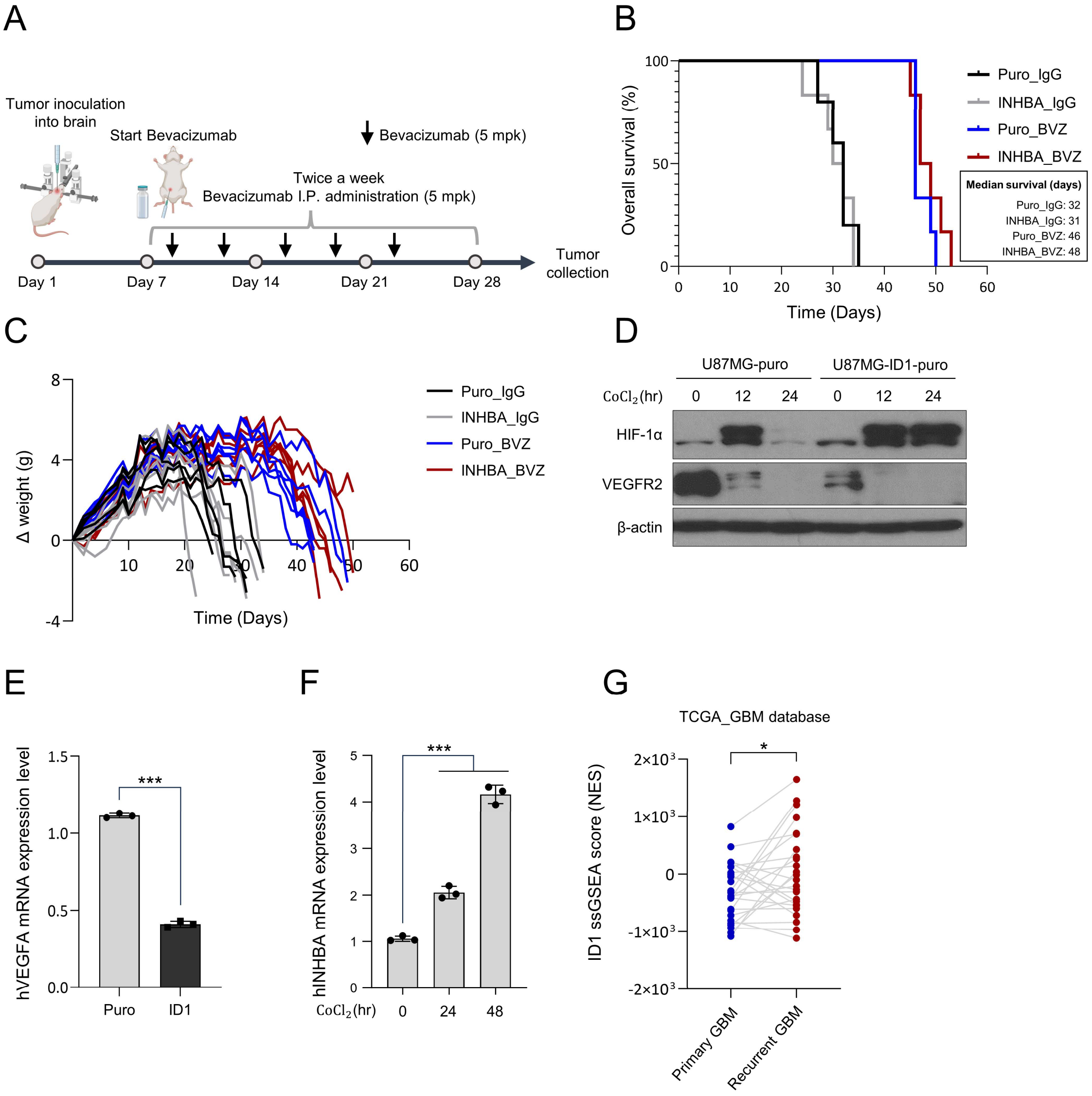
A
Tumor inoculation into brain
Start Bevacizumab
Bevacizumab (5 mpk)
Twice a week
Bevacizumab I.P. administration (5 mpk)
Day 1
Day 7
Day 14
Day 21
Day 28
Tumor collection
B
Overall survival (%)
Time (Days)
Puro_IgG
INHBA_IgG
Puro_BVZ
INHBA_BVZ
Median survival (days)
Puro_IgG: 32
INHBA_IgG: 31
Puro_BVZ: 46
INHBA_BVZ: 48
C
Δ weight (g)
Time (Days)
Puro_IgG
INHBA_IgG
Puro_BVZ
INHBA_BVZ
D
U87MG-puro
U87MG-ID1-puro
CoCl2 (hr)
0
12
24
0
12
24
HIF-1α
VEGFR2
β-actin
E
hVEGFA mRNA expression level
***
Puro
ID1
F
hINHBA mRNA expression level
***
CoCl2 (hr)
0
24
48
G
TCGA_GBM database
ID1 ssGSEA score (NES)
*
Primary GBM
Recurrent GBM

Supplemental Fig. 7 Activin A alone does not confer resistance to BVZ and clinical relevance of ID1.

(A) Experimental scheme for the treatment of the GBM intracranial graft mouse model with BVZ (5 mpk).

(B) Kaplan-Meier plots showing the survival rates of the GBM intracranial graft mouse model treated with BVZ. n = 5-6 per group. *P* values were obtained using Log-rank (Mantel-Cox) test.

(C) Bodyweight of the intracranial graft mouse model. n = 5-6 per group.

(D) Western blot analysis of HIF-1α, VEGFR2, and β-actin in U87MG-puro and U87MG-ID1-puro treated with $CoCl_2$ (300 μM).

(E) The mRNA level of *VEGFA* in U87MG-puro and U87MG-ID1-puro. ***$P < 0.001$ by unpaired t-tests.

(F) The mRNA level of *INHBA* in U87MG-ID1-puro treated with $CoCl_2$ (300 μM). ***$P < 0.001$ by unpaired t-tests.

(G) Single-sample GSEA was performed using RNA-sequencing data from ID1-overexpressing U87MG cells in patients with primary and recurrent GBM. *$P < 0.05$ by unpaired t-tests.

Supplemental Movie legends

Supplemental Movie 1: Vascular permeability in 3D-vasculogenesis microchip model.

Live-imaging showing leakage of 500 kDa-TRITC-dextran out of the lumen structure in a 3D-vasculogenesis microchip model. Scale bar, 100 µm.

Supplemental Movie 2: Activin A increases vascular permeability in 3D-vasculogenesis microchip model.

Live-imaging showing leakage of 500 kDa-TRITC-dextran out of the lumen structure in a 3D-vasculogenesis microchip model with activin A. Scale bar, 100 µm.

References for supplemental information

40. Choi SH, Park MJ, Kim H. Inhibitor of differentiation 1 in U87MG glioblastoma cells promotes HUVEC sprouting through endothelin-1. Oncol Lett. 2022;24(5):413.

41. Park CG, Choi SH, Lee SY, Eun K, Park MG, Jang J, et al. Cytoplasmic LMO2-LDB1 Complex Activates STAT3 Signaling through Interaction with gp130-JAK in Glioma Stem Cells. Cells. 2022;11(13).

42. Urup T, Staunstrup LM, Michaelsen SR, Vitting-Seerup K, Bennedbæk M, Toft A, et al. Transcriptional changes induced by bevacizumab combination therapy in responding and non-responding recurrent glioblastoma patients. BMC Cancer. 2017;17(1):278.

43. Reifenberger G, Weber RG, Riehmer V, Kaulich K, Willscher E, Wirth H, et al. Molecular characterization of long-term survivors of glioblastoma using genome- and transcriptome-wide profiling. Int J Cancer. 2014;135(8):1822-31.

44. Kwon SM, Kang SH, Park CK, Jung S, Park ES, Lee JS, et al. Recurrent Glioblastomas Reveal Molecular Subtypes Associated with Mechanistic Implications of Drug-Resistance. PLoS One. 2015;10(10):e0140528.

45. Donson AM, Birks DK, Schittone SA, Kleinschmidt-DeMasters BK, Sun DY, Hemenway MF, et al. Increased immune gene expression and immune cell infiltration in high-grade astrocytoma distinguish long-term from short-term

survivors. J Immunol. 2012;189(4):1920-7.
