# Supplementary material for: ID1high/activin Ahigh glioblastoma cells contribute to resistance to anti-angiogenesis therapy through malformed vasculature
Source: Cell Death Dis. 2024 Apr 24;15(4):292. doi: 10.1038/s41419-024-06678-7 (PMC11043395; doi:10.1038/s41419-024-06678-7)
Supplement: Supplementary file 4 — Supplementary Table [file 41419_2024_6678_MOESM4_ESM.docx]

**Supplementary Table**

**ID1^high^/activin A^high^ glioblastoma cell contributes to resistance of anti-angiogenesis therapy through malformed vasculature**

Sang-Hun Choi, Junseok Jang, Yoonji Kim, Cheol Gyu Park, Seon Yong Lee, Hyojin Kim, and Hyunggee Kim

**Supplementary Table 1:** **List of primers used in this study**

| **Gene** | **Primer** | **Sequence (5’ to 3’)** |
| --- | --- | --- |
| *CCN1* | Forward | GCTTGTTGGCGTCTTCGT |
|  | Reverse | ATTCCAGCCCCTTGGTGT |
| *CDH2* | Forward | AATGACAATGCCCCTCAAGT |
|  | Reverse | TCACACGCAGGATGGAAATA |
| *CDH5* | Forward | CCCTGCTCCAACTCCATACT |
|  | Reverse | GAACATCTGCCCCTTCTCAG |
| *CLDN5* | Forward | AGACTGAGGATTTCGCTTCC |
|  | Reverse | CCTGCCGATGGAGTAAAGAC |
| *EHD4* | Forward | CTTGGGGAGGTAGGTGACT |
|  | Reverse | CAGTGGGACGGTTCTGATTT |
| *FN1* | Forward | GGCCAGTCCTACAACCAGTA |
|  | Reverse | CTCTCGGGAATCTTCTCTGTC |
| *FSCN1* | Forward | TCATCAACCGCCCCATCATC |
|  | Reverse | TGCCCACCGTCCAGTATTTG |
| *GAPDH* | Forward | CTACACTGAGCACCAGGTGGTCTC |
|  | Reverse | GATGGATACATGACAAGGTGCGGC |
| *ID1* | Forward | GCTGCTCTACGACATGAACG |
|  | Reverse | CTCCAACTGAAGGTCCCTGA |
| *IGFBP3* | Forward | TGGACACACGCTGCATAG |
|  | Reverse | TGATACAAAGCCCAGAGATGGT |
| *INHBA* | Forward | AAAAGAAGGGCGGAGGTGAAG |
|  | Reverse | CAGCCGATGTCCTTGAAACTG |
| *JAM2* | Forward | TGATTTAGGGGGTCCAACAA |
|  | Reverse | CTCCAGCCATTAGGCATTTC |
| *LAMA4* | Forward | CAGTGTCAATGGGGAGTACC |
|  | Reverse | CTGCTTGGGTGTAACTGAGG |
| *LAMB1* | Forward | ACTGGGGAGTGCTTGAACTG |
|  | Reverse | GTCGCCATAGTAACCAGCCA |
| *NOTCH4* | Forward | TGTGAGAAAGGCTGCAACAC |
|  | Reverse | GCCATCACGATCCTTCCTTA |
| *PECAM1* | Forward | TGAGGTCAAAGGATCAGACGAC |
|  | Reverse | TGGTGGCAAGGGACTAAGGA |
| *SNAI1* | Forward | CCCAATCGGAAGCCTAACTA |
|  | Reverse | GGCTGCTGGAAGGTAAACTC |
| *SNAI2* | Forward | TGGTTGCTTCAAGGACACAT |
|  | Reverse | GCAGATGAGCCCTCAGATTT |
| *TGM2* | Forward | CAACCTGGAGCCTTTCTCTG |
|  | Reverse | GCACCTTGATGAGGTTGGAC |
| *TJP* | Forward | GCTTCATCTCCACTCCCTTACC |
|  | Reverse | TGGTGAGTAAGAGGTGGAAGGA |
| *TSPAN7* | Forward | TCTTTGGCCTGTTTGGATGCT |
|  | Reverse | TCTGCATAGCGTCCGTGTAAG |
| *VEGFA* | Forward | TGAGCTTCCTACAGCACAAC |
|  | Reverse | GAGGCTCCAGGGCATTAGA |
| *vWF* | Forward | GGGCTTCACTTACGTTCTGC |
|  | Reverse | CCTTCACTCGGACACACTCA |
| *ZEB1* | Forward | TGCACTGAGTGTGGAAAAGC |
|  | Reverse | TGGTGATGCTGAAAGAGACG |
| *ZEB2* | Forward | ACGGTATTGCCAACCCTCTG |
|  | Reverse | GGTCTGGATCGTGGCTTCTG |

1. List of qRT-PCR primers used in this study
