# Supplementary material for: ID1high/activin Ahigh glioblastoma cells contribute to resistance to anti-angiogenesis therapy through malformed vasculature
Source: Cell Death Dis. 2024 Apr 24;15(4):292. doi: 10.1038/s41419-024-06678-7 (PMC11043395; doi:10.1038/s41419-024-06678-7)

Uncropped western blot

Figure 3B

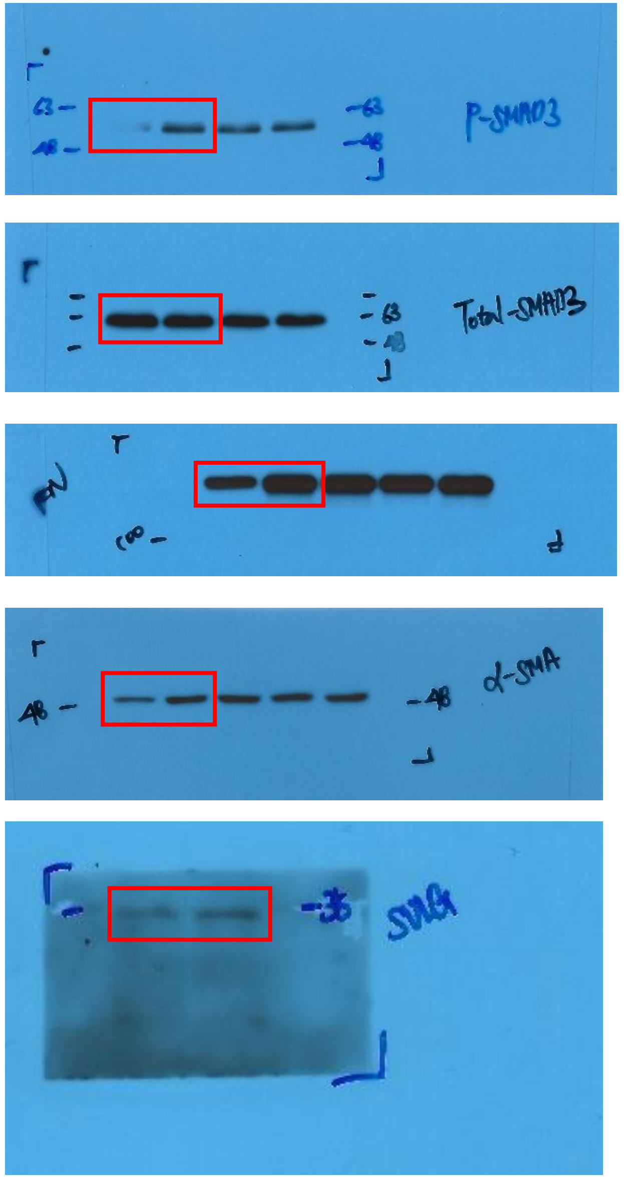

Figure 6A

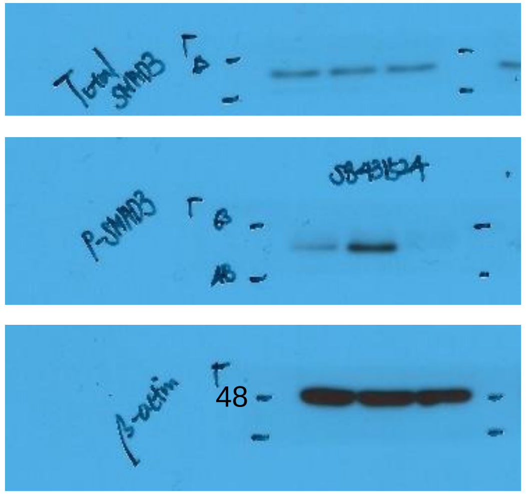

Supplementary Figure 2C

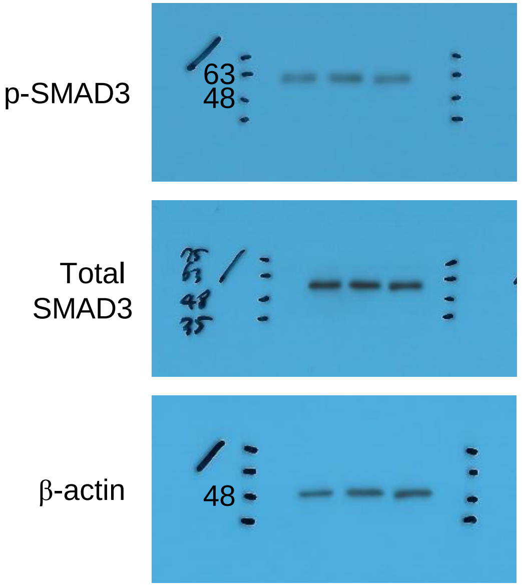

Supplementary Figure 1A

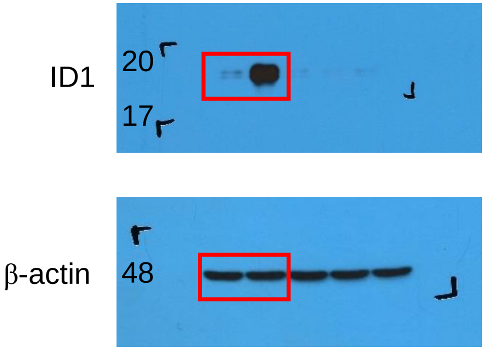

Supplementary Figure 7D

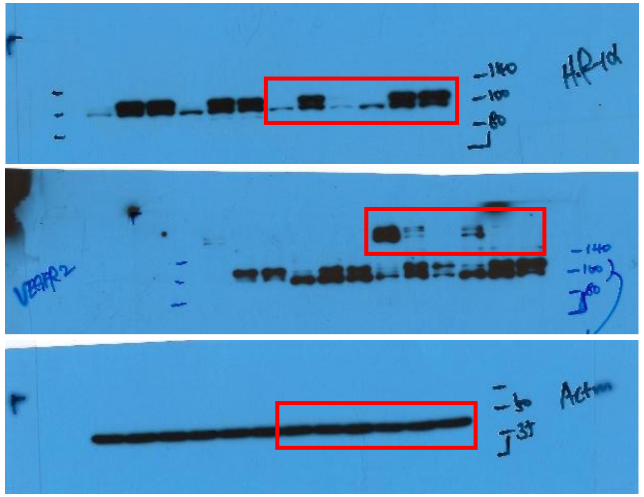

Supplement: Supplementary file 5 — Uncropped WB images [file 41419_2024_6678_MOESM5_ESM.pdf]
